# Supplementary material for: Barcoding of small extracellular vesicles with CRISPR-gRNA enables comprehensive, subpopulation-specific analysis of their biogenesis and release regulators
Source: Nat Commun. 2024 Nov 19;15:9777. doi: 10.1038/s41467-024-53736-x (PMC11577021; doi:10.1038/s41467-024-53736-x)
Supplement: Supplementary file 1 — Supplementary Information [file 41467_2024_53736_MOESM1_ESM.pdf]

Supplementary Information

Barcoding of small extracellular vesicles with CRISPR-gRNA enables comprehensive, subpopulation-specific analysis of their biogenesis/release regulators

Koki Kunitake[1,2], Tadahaya Mizuno[2], Kazuki Hattori[3], Chitose Oneyama[4], Mako Kamiya[5], Sadao Ota[3], Yasuteru Urano[1,2], Ryosuke Kojima[1,6,7]*

[1] Graduate School of Medicine, The University of Tokyo, Tokyo 113-0033, Japan.

[2] Graduate School of Pharmaceutical Sciences, The University of Tokyo, 113-0033 Tokyo, Japan.

[3] Research Center for Advanced Science and Technology, The University of Tokyo, 153-8904 Tokyo, Japan.

[4] Division of Cancer Cell Regulation, Aichi Cancer Center Research Institute, 464-8681 Nagoya, Japan

[5] Department of Life Science and Technology, Institute of Science Tokyo, 226-8501 Kanagawa, Japan.

[6] PRESTO, Japan Science and Technology Agency, Kawaguchi, Saitama, 332-0012, Japan

[7] FOREST, Japan Science and Technology Agency, Kawaguchi, Saitama, 332-0012, Japan

*Email: kojima@m.u-tokyo.ac.jp

Inventory

Supplementary Table 1. Plasmids used in this study 4
Supplementary Table 2. Oligonucleotides used in this study 6
Supplementary Table 3. Barcode information of NGS primers 8
Supplementary Table 4. Sources of siRNA used in this study 9

Supplementary Fig. 1. Comparison of pooled CRISPR screening and CIBER screening 10
Supplementary Fig. 2. Characterization of sEVs 11
Supplementary Fig. 3. Description and performance of MS2-based gRNA loading system 12
Supplementary Fig. 4. Results of nanoparticle tracking analysis (NTA) for sEVs isolated from culture media (CM) of HEK293T cells expressing each component 12
Supplementary Fig. 5. Confirmation that co-expression of Cas9 has little effect on coverage of gRNAs in sEVs....13
Supplementary Fig. 6. Confirmation that CD63-dCas9 expression has little effect on Cas9-induced gene KO..... 13
Supplementary Fig. 7. gRNA libraries used in this study and their barcoding performance in sEVs 14
Supplementary Fig. 8. Evaluation of the effect of cellular gRNA level on gRNA amount in sEVs 15
Supplementary Fig. 9. Raw measurement of LI and protein amount used to generate Fig. 3b and c 15
Supplementary Fig. 10. Concentration of sEVs released from HEK293T cells treated with C75 (FASN inhibitor) or GSK-A1 (PI4KA inhibitor) 16
Supplementary Fig. 11. Confirmation of gene knockdown by siRNA used for validation in Fig. 3f 16
Supplementary Fig. 12. CD63-nluc reporter assay for YKT6 (*z*-RE = -3.40) by siRNA 17
Supplementary Fig. 13. Matching of the hit genes of CD63-CIBER and top 1,000 sEVs-associated proteins compiled from the VesiclePedia database (related to Fig. 3g) 18
Supplementary Fig. 14. Connection of the hit genes in CD63-CIBER with known sEV release regulators (ARF1, HGS, PDCD6IP, RAB5A, RAB9A, SEC22A, SMPD3, SNAP29, STX2, STX7, STX16, STX18, USE1 and VAMP7) analyzed by STRING 19
Supplementary Fig. 15. Results of small-scale CD63-CIBER screening with SH-SY5Y cells and comparison with the results for HEK293T cells 20
Supplementary Fig. 16. Results of CD9-CIBER screening 21
Supplementary Fig. 17. *z*-REs of ESCRT genes obtained from CD63-CIBER and CD9-CIBER screening 22
Supplementary Fig. 18. Detailed analysis of OxPhos terms and their annotated genes 23
Supplementary Fig. 19. Highlighted scatter plot of genes annotated to either of GO terms on Fig. 4c, overlaid on the comparative scatter plot of Fig. 4a with dashed diagonal line 24
Supplementary Fig. 20. Orthogonal validation of the effect of rotenone and concanamycin A 25
Supplementary Fig. 21. CD63-nluc and CD9-nluc reporter assays for *NDUFS1* (*z*-RE = -2.71 in CD63-CIBER, -1.29 in CD9-CIBER) using siRNAs 25
Supplementary Fig. 22. Subcellular localization study of CD63 and CD9 26

Supplementary Fig. 23. Confirmation of lysosomal activity upon treatment with inhibitors 27
Supplementary Fig. 24. Term frequency - inverse document frequency (TF-IDF) analysis of hit terms 28
Supplementary Fig. 25. Flow cytometry analysis of cell cycle arrest by dinaciclib 29
Supplementary Fig. 26. Comparison of sequencing results of CIBER screening and a previous study 30
Supplementary Fig. 27. Volcano plot of enrichment score calculated via GSEAPreranked with z-FC_{sEVs} as the query array 31
Supplementary Fig. 28. Time-dependent change of RNA expression profile after RAB27A and PDCD6IP KO in HEK293T cells 32
Supplementary Fig. 29. Subcellular localization study of CD63-dCas9 and CD9-dCas9 in HEK293T cells (compared with CD63/CD9-sfGFP) 34
Supplementary Fig. 30. *z*-RE scores of genes essential for cell survival (Essential genes) and genes not essential for cell survival (Nonessential genes) in CD63-CIBER and CD9-CIBER in HEK293T cells. 36
Supplementary Fig. 31. gRNA abundance in sEVs isolated from culture media of HEK293T or HEK293T cells expressing dCas9-fused EV marker (TSG101, PTGFRNΔ687 and ALIX) stably transduced with gRNA using lentivirus 37
Supplementary Fig. 32. Data showing the applicability of the sEV-barcoding system for cell-free CRISPR screening 38

Supplementary references 39

Uncropped Scans 41

Plasmid	Description and Cloning Strategy	Reference /Source
**pSBbi-GH**	Empty Sleeping Beauty transposon with constitutive bi-directional promoter, one side: SfiI cloning site for GOI (driven by EF1α promoter), other side: EGFP and hygromycin resistance gene (driven by RPBSA promoter)	Kowarz *et al*, addgene #60514[1]
**pSBbi-RB**	Empty Sleeping Beauty transposon with constitutive bi-directional promoter, one side: SfiI cloning site for GOI (driven by EF1α promoter), other side: dTomato and blasticidin resistance gene (driven by RPBSA promoter)	Kowarz *et al*, addgene #60522[1]
**pSBbi-Hyg**	Empty Sleeping Beauty transposon with constitutive bi-directional promoter, one side: SfiI cloning site for GOI (driven by EF1α promoter), other side: hygromycin resistance gene (driven by RPBSA promoter)	Kowarz *et al*, addgene #60524[1]
**pCRISPRia-v2**	CRISPRi/a V2 library parental plasmid which expresses gRNA from U6 promoter and tagBFP and puromycin resistance from EF1α promoter.	Horlbeck *et al*, addgene #84832[2]
**pKK47**	Constitutive expression of CD63-MS2. CD63-MS2 was PCR-amplified from pSA_Exo010 (unpublished, encoding MS2 reported by Wroblewska et al[3]) from using oKK29 and oKK103, digested with SfiI and cloned into the corresponding sites (SfiI) of pSBbi-GH (addgene #60514[1]).	This work
**pKK49**	Constitutive expression of mock MS2-gRNA for lentiviral transduction. gRNA scaffold bearing MS2 coat protein-binding motif was PCR-amplified from sgRNA(MS2) cloning backbone (addgene #61424[4]) using oKK109 and oKK105, digested with BlpI/XhoI and cloned into the corresponding sites (BlpI/XhoI) of pCRISPRia-v2 (addgene #84832[2]).	This work
**pKK59**	Constitutive expression of CD63 with EFGGGGSGTIS linker at the C-terminal. CD63 was PCR-amplified from pDB30[5] using oKK127 and oKK128, digested with SfiI and cloned into the corresponding sites (SfiI) of pSBbi-GH (addgene #60514[1]). The GTIS sequence in the linker can be digested by KpnI-HF/NheI-HF to allow insertion of the gene to be fused to CD63	This work
**pKK60**	Constitutive expression of CD63-dCas9. dCas9 was PCR-amplified from lenti-EF1α-dCas9-KRAB-Puro (addgene #99372[6]) using oKK129 and oKK130 (excluding N-terminal nuclear localization signal (NLS), KRPAATKKAGQAKKKK), digested with KpnI-HF/NheI-HF and cloned into the corresponding sites (KpnI-HF/NheI-HF) of pKK59.	This work
**pRK300**	Constitutive expression of Cas9. Cas9 was PCR-amplified from lentiCas9-Blast (addgene #52962[7]) using oRyo004 and oRyo005, digested with SfiI and cloned into the corresponding sites (SfiI) of pSBbi-RB (addgene #60522[1]).	This work
**pKK89**	Constitutive expression of non-targeting gRNA (spacer sequence; GAAAGAGGGTCCCCCTGTAG). The non-targeting spacer was inserted by annealing oKK206 and oKK207 and cloned into pCRISPRia-v2 (addgene #84832[2]) which was digested with BlpI/BstXI to remove dummy spacer. The spacer sequence was selected from the Bassik human deletion library[8].	This work
**pKK90**	Constitutive expression of non-targeting gRNA (gRNA #1) (spacer sequence; GAACAAGTTCCAATTTGTAT).oKK208 and oKK209 were inserted to pCRISPRia-v2 (addgene #84832[2]) in the same way as pKK89. The spacer sequence was selected from the Bassik human deletion library[8].	This work
**pKK209**	Constitutive expression of BFP-targeting gRNA (gRNA #2) (spacer sequence; GCACATGAAGCTGTACATGG). oKK531 and oKK532 were inserted to pCRISPRia-v2 (addgene #84832[2]) in the same way as pKK89. The spacer sequence was designed using CHOPCHOP[9].	This work
**pKK210**	Constitutive expression of BFP-targeting gRNA (spacer sequence; GGCGAAGGCAAGCCCTACGA). oKK533 and oKK534 were inserted to pCRISPRia-v2 (addgene #84832[2]) in the same way as pKK89. The spacer sequence was designed using CHOPCHOP[9].	This work
**pKK104**	Constitutive expression of CD9 with EFGGGGSGTIS linker at the C-terminal. CD9 was PCR-amplified from pDB96[5] (P_{hCMV}-CD9-nluc-pA_{bGH}) using oKK620 and oKK621, digested with SfiI and cloned into the corresponding sites (SfiI) of pSBbi-GH (addgene #60514[2]). The GTIS sequence in the linker can be digested by KpnI-HF/NheI-HF to allow insertion of the gene to be fused to CD9.	This work

**pKK106**	Constitutive expression of CD9-dCas9. dCas9-encoding DNA fragment was collected from pKK60 digested with KpnI-HF/NheI-HF followed by agarose gel purification and inserted into the corresponding sites (KpnI-HF/NheI-HF) of pKK104.	This work
**pKK108**	Constitutive expression of CD63-nluc. nluc was PCR-amplified from pDB30[5] (P_{hCMV}-CD63-nluc-pA_{bGH}) using oKK236 and oKK237, digested with KpnI-HF/NheI-HF and cloned into the corresponding sites (KpnI-HF/NheI-HF) of pKK59.	This work
**pKK147**	Constitutive expression of CD9-nluc. nluc-encoding DNA fragment was collected from pKK108 digested with KpnI-HF/NheI-HF and inserted into the corresponding sites (KpnI-HF/NheI-HF) of pKK104.	This work
**pKK148**	Constitutive expression of CD63 with EFGGGGSGTIS linker at C-terminal. CD63-linker-encoding DNA fragment was collected from pKK59 digested with SfiI followed by agarose gel purification and inserted into the corresponding sites (SfiI) of pSBbi-Hyg (addgene #60524[2]).	This work
**pKK149**	Constitutive expression of CD9 with EFGGGGSGTIS linker at the C-terminal. CD9-linker-encoding DNA fragment was collected from pKK104 digested with SfiI followed by agarose gel purification and inserted into the corresponding sites (SfiI) of pSBbi-Hyg (addgene #60524[2]).	This work
**pKK150**	Constitutive expression of CD63-sfGFP. sfGFP was PCR-amplified from pHRdSV40-scFv-GCN4-sfGFP-VP64-GB1-NLS (addgene #60904[10]) using oKK334 and oKK335, digested with KpnI-HF/NheI-HF and cloned into the corresponding sites (KpnI-HF/NheI-HF) of pKK148.	This work
**pRK397**	Constitutive expression of CD63-mScarlet. mScarlet was PCR-amplified from pmScarlet_C1 (addgene #85042[11]) using oRK164 and oRK165, digested with KpnI-HF/NheI-HF and cloned into the corresponding sites (KpnI-HF/NheI-HF) of pKK148.	This work
**pKK151**	Constitutive expression of CD9-sfGFP. sfGFP was PCR-amplified from pHRdSV40-scFv-GCN4-sfGFP-VP64-GB1-NLS (addgene #60904[10]) using oKK334 and oKK335, digested with KpnI-HF/NheI-HF and cloned into the corresponding sites (KpnI-HF/NheI-HF) of pKK149.	This work
**pKK180**	Constitutive expression of CD63-dCas9 (co-expressing dTomato, not EGFP unlike pKK60). CD63-dCas9-encoding DNA fragment was collected from pKK60 digested with SfiI followed by agarose gel purification and inserted into the corresponding sites (SfiI) of pSBbi-RB (addgene #60522[1])	This work
**pKK272**	Constitutive expression of TSG101-dCas9. This plasmid was constructed by a stepwise cloning by normal restriction cloning using pKK60 (CD63-dCas9) as a backbone. The CD63-encoding region was replaced by TSG101-encoding fragment derived from pLNCX2-mEGFP-TSG101 (addgene #116925).	This work
**pKK273**	Constitutive expression of ALIX-dCas9. This plasmid was constructed by a stepwise cloning by normal restriction cloning using pKK60 (CD63-dCas9) as a backbone. The CD63-encoding region was replaced by ALIX-encoding fragment derived from pCINeoFlag ALIX (addgene #89859).	This work
**pKK274**	Constitutive expression of PTGFRNΔ687[12]-dCas9. This plasmid was constructed by a stepwise cloning by normal restriction cloning using pKK60 (CD63-dCas9) as a backbone. The CD63-encoding region was replaced by PTGFRNΔ687-encoding fragment derived from synthesized DNA.	This work
**pKK275**	Constitutive expression of RAB27A-targeting gRNA (spacer sequence; GTGGGCCGGATGGAGCCAC). oKK666 and oKK667 were inserted to pCRISPRia-v2 (addgene #84832[2]) in the same way as pKK89.	This work
**pKK278**	Constitutive expression of PDCD6IP-targeting gRNA (spacer sequence; GGTCGGCTCTCGACTTA). oKK672 and oKK673 were inserted to pCRISPRia-v2 (addgene #84832[2]) in the same way as pKK89.	This work

Supplementary Table 1. Plasmids used in this study.

Name	Usage	Sequence (5' -> 3')
**Oligo #1**	gRNA detection by qPCR Fw	AGCTAAGCTGGAAACAGCA
**Oligo #2**	gRNA detection by qPCR Rev	CGACTCGGTGCCACTTT
**Oligo #3**	gRNA detection probe	5'-FAM-AAGGCTAGT/ZEN/CCGTTATCAACTTGAA-3'-IABKFQ
**oKK145**	RT primer for gRNA	TTTTTCAAGTTGATAACGGACTAGCC
**oKK147**	PCR Rev for gRNA	CCTCTCTATGGGCAGTCGGTGATGACTAGCCTTATTTAAACTTGCTATG
**Oligo #4**	RT primer for MS2-gRNA	AAAGCACCGACTCGGTGCCAC
**Oligo #5**	PCR Rev for MS2-gRNA	CCTCTCTATGGGCAGTCGGTGATGCCAAGTTGATAACGGACTAGCCTT
**LNA-TSO**	template switching oligo	AAGCAGTGGTATCAACGCAGAGTACrGrG+G
**oKK120**	qPCR Fw for NGS sample	CCATCTCATCCCTGCGTGTCTCC
**oKK121**	qPCR Rev for NGS sample	CCTCTCTATGGGCAGTCGGTGAT
**oKK29**	subcloning pKK47 Fw	ATTAGGCCTCTGAGGCCACCATGGCGGTGGAAGGAGGAATGAAATGTGTGAAG
**oKK103**	subcloning pKK47 Rev	ATTAGGCCTGACAGGCCTTACGCGTAGATGCCGGAGTTTGCTGCG
**oKK109**	subcloning pKK49 Fw	GTCTTCGAGAAGACCTGTTTAAGAGCTAAGCCAAC
**oKK105**	subcloning pKK49 Rev	ATTACTCGAGAAAAAAAGCACCGACTCGGTGCCACTTGG
**oKK127**	subcloning pKK59 Fw	ATTAGGCCTCTGAGGCCAGCTTGCCACCATGGCGGTG
**oKK128**	subcloning pKK59 Rev	ATTAGGCCTGACAGGCCGCTAGCTAATGGTACCGGACCCGCCTCCGCCGAATTC
**oKK129**	subcloning pKK60 Fw	ATTAGGTACCGACAAGAAGTACAGCATCGGCCTG
**oKK130**	subcloning pKK60 Rev	ATTAGCTAGCCTAGTCGCCTCCCAGCTGAGACAG
**oRyo004**	subcloning pRK300 Fw	ATTAGGCCTCTGAGGCCACCATGGACAAGAAGTACAGCATCGG
**oRyo005**	subcloning pRK300 Rev	TAATGGCCTGACAGGCCTACTTATCGTCATCGTCTTTGTAATCTTTCTTCTTCTTAGCCTGTCCAG
**oKK206**	subcloning pKK89 Fw	TTGGAAAGAGGGTCCCCCTGTAGGTTTAAGAGC
**oKK207**	subcloning pKK89 Rev	TTAGCTCTTAAACCTACAGGGGGACCCTCTTTCCAACAAG
**oKK208**	subcloning pKK90 Fw	TTGGAACAAGTTCCAATTTGTATGTTTAAGAGC
**oKK209**	subcloning pKK90 Rev	TTAGCTCTTAAACATACAAATTGGAACTTGTTCCAACAAG
**oKK531**	subcloning pKK209 Fw	TTGGCACATGAAGCTGTACATGGGTTTAAGAGC
**oKK532**	subcloning pKK209 Rev	TTAGCTCTTAAACCCATGTACAGCTTCATGTGCCAACAAG
**oKK533**	subcloning pKK210 Fw	TTGGGCGAAGGCAAGCCCTACGAGTTTAAGAGC
**oKK534**	subcloning pKK210 Rev	TTAGCTCTTAAACTCGTAGGGCTTGCCTTCGCCCAACAAG
**oKK600**	subcloning pKK104 Fw	ATTAGGCCTCTGAGGCCACCATGCCGGTCAAAGGAGGCACCAAG
**oKK621**	subcloning pKK104 Rev	ATTAGGCCTGACAGGCCGCTAGCTAATGGTACCGGACCCGCCTCCGCCGAATTCCAAGACCATCTCGCGGTTCCTGCG
**oKK236**	subcloning pKK108 Fw	ATTAGGTACCATGGTCTTCACACTCGAAGATTTCG
**oKK237**	subcloning pKK108 Rev	ATTAGCTAGCTTACGCCAGAATGCGTTCGC
**oRK164**	subcloning pRK397 Fw	ATTAGGTACCGTGAGCAAGGGCGAGGCAGT
**oRK165**	subcloning pRK397 Rev	ATTAGCTAGCCTACTTGTACAGCTCGTCCATGCCG

**oKK334**	subcloning pKK151 Fw	ATTAGGTACCAGCAAAGGAGAAGAACTTTTCAC
**oKK335**	subcloning pKK151 Rev	ATTAGCTAGCCTATTTGTAGAGCTCATCCATGCC
**oKK666**	subcloning pKK275 Fw	TTGGTGGGCCGGATGGAGCCACGTTTAAGAGC
**oKK667**	subcloning pKK275 Rev	TTAGCTCTTAAACGTGGCTCCATCCGGCCCACCAA CAAG
**oKK672**	subcloning pKK278 Fw	TTGGGTCGGCTCTCGACTTAGTTTAAGAGC
**oKK673**	subcloning pKK278 Rev	TTAGCTCTTAAACTAAGTCGAGAGCCGACCCAACA AG
**oKK370**	qPCR OSBP Fw	GATCCATCAGGAAAAGTCCAC
**oKK371**	qPCR OSBP Rev	CAGTGCCACTTTCCCAAGCA
**oKK362**	qPCR FASN Fw	CGCGTGGCCGGCTACTCCTAC
**oKK363**	qPCR FASN Rev	CGGCTGCCACACGCTCCTCT
**oKK382**	qPCR TMED10 Fw	GAGATGCGTGATACCAACGA
**oKK383**	qPCR TMED10 Rev	TTCTTGGCCTTGAAGAAGCG
**oKK368**	qPCR GOLGA2 Fw	ACGGATCAGTTGGAAGAAGAAA
**oKK369**	qPCR GOLGA2 Rev	GGATCCCTATGGTCTGAATGTG
**oKK585**	qPCR NDUFS1 Fw	GGAAGAACCCTCCCAAGGTG
**oKK586**	qPCR NDUFS1 Rev	TGCTGAGCTCTACCCTCAGT
**oKK388**	qPCR PTPN23 Fw	GCCAGCTGTGAAGAAGTTTGT
**oKK389**	qPCR PTPN23 Rev	ACAGCCCTCAAAGTC TCGTG
**oKK583**	qPCR KIAA1109 Fw	GGTTTCCTGCTGTTCAGCTTGC
**oKK584**	qPCR KIAA1109 Rev	TCTGCTCTGAGGCAGTCCAAAG
**oKK402**	qPCR CAB39 Fw	CACGTTTTTAAGGTGTTTGTAGCC
**oKK403**	qPCR CAB39 Rev	ATCCTCCGTCCTGTCGTTCTG
**oKK392**	qPCR VPS28 Fw	TTGTTCCCAGGGGCTCCTAT
**oKK393**	qPCR VPS28 Rev	ATCTTCCCGACCGCGAG
**oKK376**	qPCR PI4KA Fw	GCCTGGAGCATCTCTCCCTA
**oKK377**	qPCR PI4KA Rev	AGGCACATCACTAACGGCTC
**oKK563**	qPCR COG3 Fw	CACTGTTGCAGAGTTAACCAGCC
**oKK564**	qPCR COG3 Rev	GGTGTTCATCCTGGCAGACATG
**oKK567**	qPCR COPG1 Fw	GCAACACGCCGTCCTTATG
**oKK568**	qPCR COPG1 Rev	CACTGTGAACTCGCAGTCCT
**oKK573**	qPCR YKT6 Fw	CTGTTAGAGCGAGGTGAGAAGC
**oKK574**	qPCR YKT6 Rev	GATGGTGCCATTCCAGCATTGG
**oKK414**	qPCR VPS25 Fw	ACAACGTCAAGCTACAGCGA
**oKK415**	qPCR VPS25 Rev	AGAAGAACTTGACGCCTCGG
**GAPDH 2F**	qPCR GAPDH Fw	TCCCTGAGCTGAACGGGAAG
**GAPDH 2R**	qPCR GAPDH Rev	GGAGGAGTGGGTGTCGCTGT
**Oligo #6-#17***	PCR Fw for gRNA	CCATCTCATCCCTGCGTGTCTCCGACTCAG(**BCD**)AAGC AGTGGTATCAACGCAGAGT
**Oligo #18**	PCR Fw for MS2-gRNA library	CCATCTCATCCCTGCGTGTCTCCGACTCAGTACCAAGA TCGATGCACAAAAGGAAACTCACCCT

Supplementary Table 2. Oligonucleotides used in this study. The barcode sequences (BCD) of Oligo #6-#17 are listed in Supplementary Table 3. rG, Riboguanosine. +G, Locked guanosine.

Barcode sequences

Name	BCD (5' -> 3')	Name	BCD (5' -> 3')
**Oligo #6**	CTGCAAGTTCGAT	**Oligo #12**	TAGGTGGTTCGAT
**Oligo #7**	TTCGTGATTCGAT	**Oligo #13**	TCTAACGGACGAT
**Oligo #8**	TTCCGATAACGAT	**Oligo #14**	TGCCACGAACGAT
**Oligo #9**	TGAGCGGAACGAT	**Oligo #15**	AACCTCATTCGAT
**Oligo #10**	CTGACCGAACGAT	**Oligo #16**	CCTGAGATACGAT
**Oligo #11**	TCCTCGAATCGAT	**Oligo #17**	TTACAACCTCGAT

Correspondence table of NGS samples and oligos used for tagging PCR

Sample	CD63_CIBER	CD9_CIBER
**ACOC_cell_rep. 1_Cas9+**	Oligo #14	Oligo #14
**ACOC_cell_rep. 1_Cas9-**	Oligo #15	Oligo #15
**ACOC_cell_rep. 2_Cas9+**	Oligo #16	Oligo #16
**ACOC_cell_rep. 2_Cas9-**	Oligo #17	Oligo #17
**ACOC_sEVs_rep. 1_Cas9+**	Oligo #14	Oligo #14
**ACOC_sEVs_rep. 1_Cas9-**	Oligo #15	Oligo #15
**ACOC_sEVs_rep. 2_Cas9+**	Oligo #16	Oligo #16
**ACOC_sEVs_rep. 2_Cas9-**	Oligo #17	Oligo #17
**DTKP_cell_rep. 1_Cas9+**	Oligo #6	Oligo #14
**DTKP_cell_rep. 1_Cas9-**	Oligo #7	Oligo #15
**DTKP_cell_rep. 2_Cas9+**	Oligo #8	Oligo #16
**DTKP_cell_rep. 2_Cas9-**	Oligo #9	Oligo #17
**DTKP_sEVs_rep. 1_Cas9+**	Oligo #10	Oligo #14
**DTKP_sEVs_rep. 1_Cas9-**	Oligo #11	Oligo #15
**DTKP_sEVs_rep. 2_Cas9+**	Oligo #12	Oligo #16
**DTKP_sEVs_rep. 2_Cas9-**	Oligo #13	Oligo #17
**PROT_cell_rep. 1_Cas9+**	Oligo #14	Oligo #14
**PROT_cell_rep. 1_Cas9-**	Oligo #15	Oligo #15
**PROT_cell_rep. 2_Cas9+**	Oligo #16	Oligo #16
**PROT_cell_rep. 2_Cas9-**	Oligo #17	Oligo #17
**PROT_sEVs_rep. 1_Cas9+**	Oligo #14	Oligo #14
**PROT_sEVs_rep. 1_Cas9-**	Oligo #15	Oligo #15
**PROT_sEVs_rep. 2_Cas9+**	Oligo #16	Oligo #16
**PROT_sEVs_rep. 2_Cas9-**	Oligo #17	Oligo #17
**TMMO_cell_rep. 1_Cas9+**	Oligo #14	Oligo #14
**TMMO_cell_rep. 1_Cas9-**	Oligo #15	Oligo #15
**TMMO_cell_rep. 2_Cas9+**	Oligo #16	Oligo #16
**TMMO_cell_rep. 2_Cas9-**	Oligo #17	Oligo #17
**TMMO_sEVs_rep. 1_Cas9+**	Oligo #14	Oligo #14
**TMMO_sEVs_rep. 1_Cas9-**	Oligo #15	Oligo #15
**TMMO_sEVs_rep. 2_Cas9+**	Oligo #16	Oligo #16
**TMMO_sEVs_rep. 2_Cas9-**	Oligo #17	Oligo #17

Supplementary Table 3. Barcode information of NGS primers.

Name	Source	Product name
**siOSBP#1**	iDT	DsiRNA, 2 nmol (hs.Ri.OSBP.13.1)
**siOSBP#2**	iDT	DsiRNA, 2 nmol (hs.Ri.OSBP.13.2)
**siOSBP#3**	iDT	DsiRNA, 2 nmol (hs.Ri.OSBP.13.3)
**siTMED10#1**	iDT	DsiRNA, 2 nmol (hs.Ri.TMED10.13.1)
**siTMED10#2**	iDT	DsiRNA, 2 nmol (hs.Ri.TMED10.13.2)
**siTMED10#3**	iDT	DsiRNA, 2 nmol (hs.Ri.TMED10.13.3)
**siGOLGA2#1**	iDT	DsiRNA, 2 nmol (hs.Ri.GOLGA2.13.1)
**siGOLGA2#2**	iDT	DsiRNA, 2 nmol (hs.Ri.GOLGA2.13.2)
**siGOLGA2#3**	iDT	DsiRNA, 2 nmol (hs.Ri.GOLGA2.13.3)
**siCtrl for iDT siRNA**	iDT	Negative Control DsiRNA, 1 nmol
**siFASN#1**	BIONEER	FASN, GENE_ID 2194, AccuTarget Genome-wide Predesigned siRNA #1
**siFASN#2**	BIONEER	FASN, GENE_ID 2194, AccuTarget Genome-wide Predesigned siRNA #2
**siFASN#3**	BIONEER	FASN, GENE_ID 2194, AccuTarget Genome-wide Predesigned siRNA #3
**siPTPN23#1**	BIONEER	PTPN23, GENE_ID 25930, AccuTarget Genome-wide Predesigned siRNA #1
**siPTPN23#2**	BIONEER	PTPN23, GENE_ID 25930, AccuTarget Genome-wide Predesigned siRNA #2
**siPTPN23#3**	BIONEER	PTPN23, GENE_ID 25930, AccuTarget Genome-wide Predesigned siRNA #3
**siCAB39#1**	BIONEER	CAB39, GENE_ID 51719, AccuTarget Genome-wide Predesigned siRNA #1
**siCAB39#2**	BIONEER	CAB39, GENE_ID 51719, AccuTarget Genome-wide Predesigned siRNA #2
**siCAB39#3**	BIONEER	CAB39, GENE_ID 51719, AccuTarget Genome-wide Predesigned siRNA #3
**siVPS28#1**	BIONEER	VPS28, GENE_ID 51160, AccuTarget Genome-wide Predesigned siRNA #1
**siVPS28#2**	BIONEER	VPS28, GENE_ID 51160, AccuTarget Genome-wide Predesigned siRNA #2
**siVPS28#3**	BIONEER	VPS28, GENE_ID 51160, AccuTarget Genome-wide Predesigned siRNA #3
**siPI4KA#1**	BIONEER	PI4KA, GENE_ID 5297, AccuTarget Genome-wide Predesigned siRNA #1
**siPI4KA#2**	BIONEER	PI4KA, GENE_ID 5297, AccuTarget Genome-wide Predesigned siRNA #2
**siPI4KA#3**	BIONEER	PI4KA, GENE_ID 5297, AccuTarget Genome-wide Predesigned siRNA #3
**siCtrl for BIONEER siRNA**	BIONEER	AccuTarget Negative Control siRNA
**siNDUFS1**	siTOOLs Biotech	siPOOL targeting human gene NDUFS1
**siCtrl for siNDFS1**	siTOOLs Biotech	Negative control siPOOL

Supplementary Table 4. Sources of siRNA used in this study.

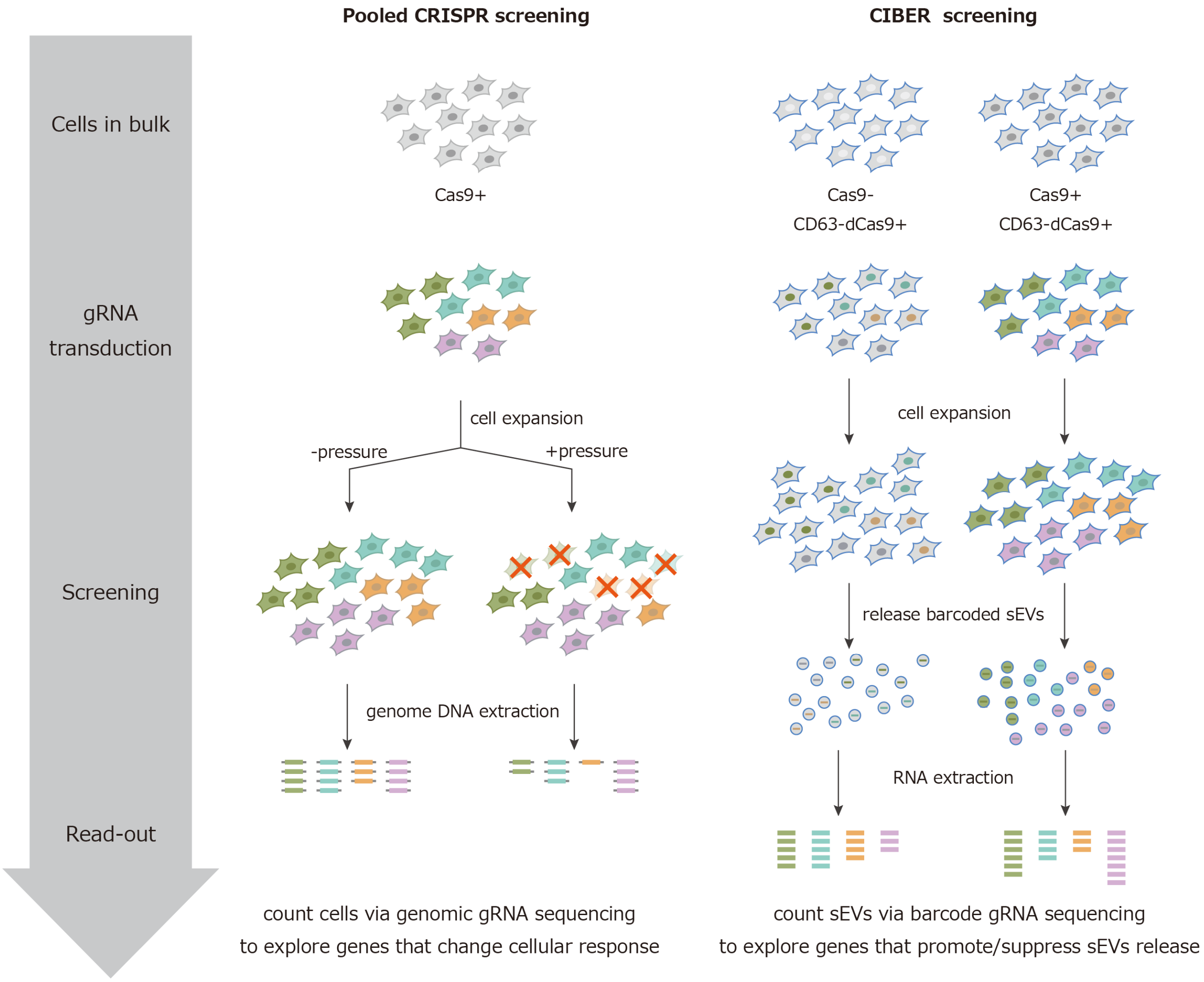

Supplementary Figure 1 | Comparison of conventional pooled CRISPR screening and CIBER screening. In CIBER screening, transcribed gRNA is used as a barcode while the gRNA expression cassette in the genome is used as a barcode in normal CRISPR screening.

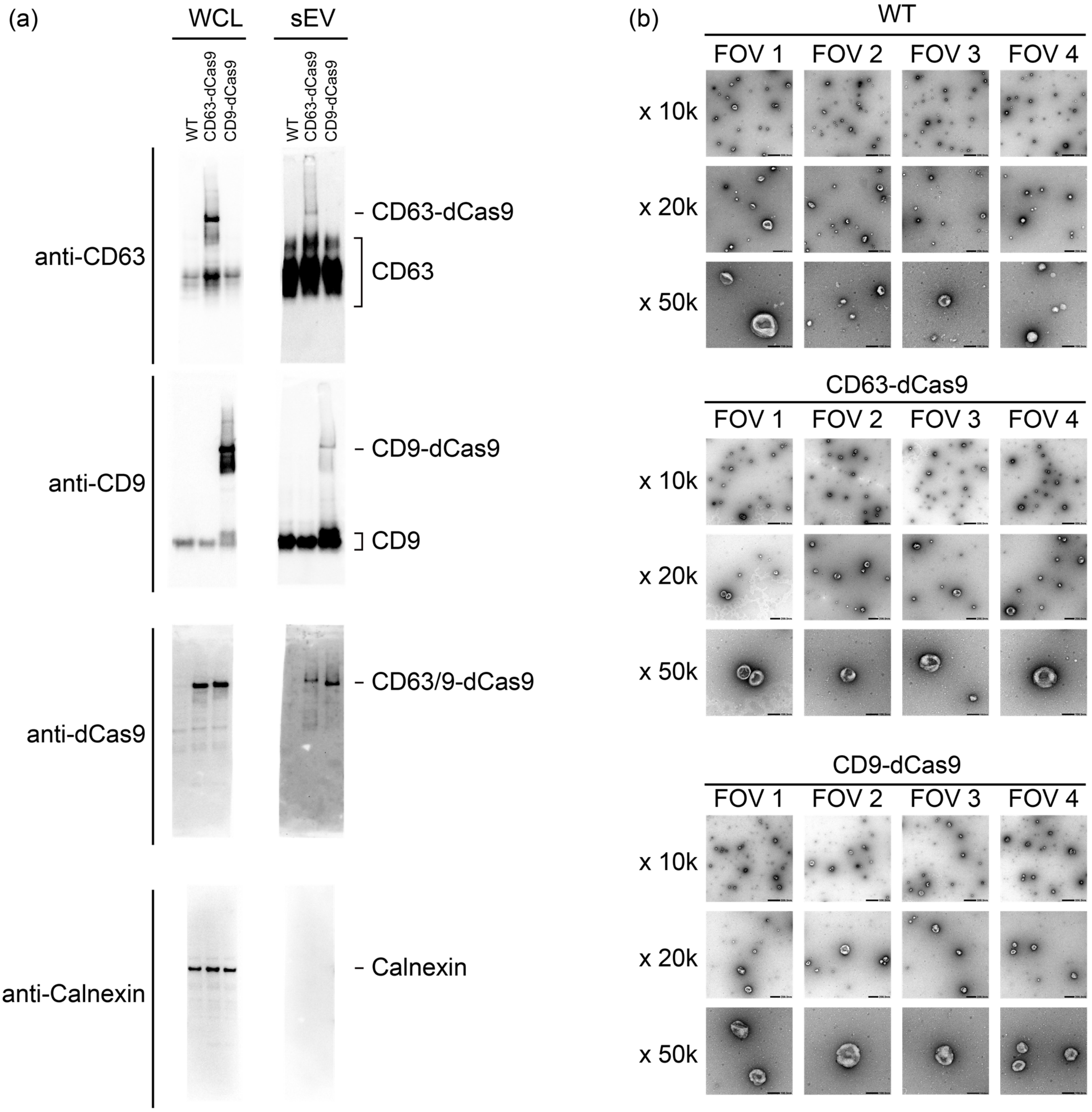

Supplementary Figure 2 | Characterization of sEVs. (a) Western blot analysis with whole cell lysate (WCL) and corresponding sEVs pellet obtained from WT HEK293T or HEK293T stably expressing CD63-dCas9 or CD9-dCas9. SDS-PAGE was conducted with 10 µg of WCL or 0.5 µg of sEV (2-3 x 10^9 particles), and the separated proteins in the gel were transferred to PVDF membrane. Two sEV marker proteins (CD63 and CD9), dCas9 fused to CD63 or CD9 and negative control (calnexin) were detected by chemiluminescence using an iBright FL5000 Imaging System. (b) Transmission electron microscopy images of sEVs negatively stained with uranium acetate. Four different fields of view (FOV) with 3 increasing magnifications for each sample were visualized using JEM-1400. Scale bar: 500, 200, 100 nm for x 10k, x 20k, and x50 k, respectively.

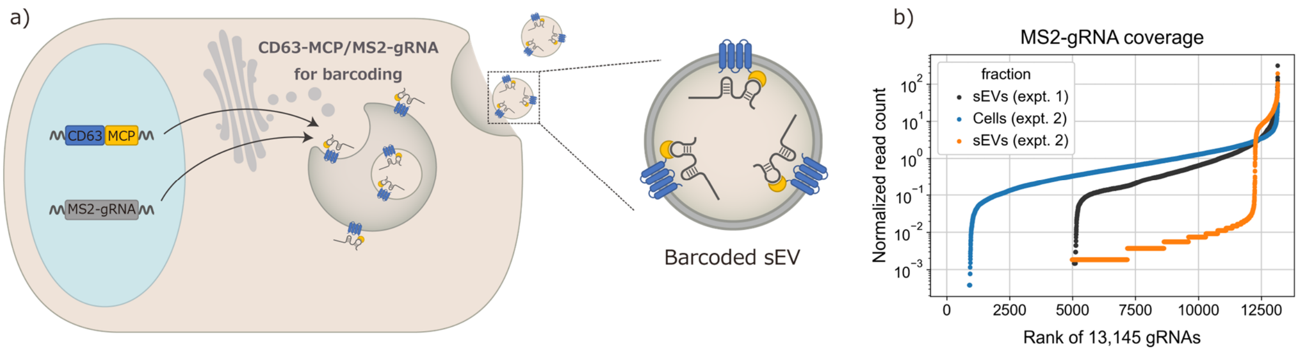

Supplementary Figure 3 | Description and performance of MS2-based gRNA loading system. (a) Strategy to barcode sEVs using CD63-MS2 coat protein (MCP) and gRNAs bearing MCP-binding motif (MS2-gRNA). Transcribed MS2-gRNA binds to CD63-fused MCP and is actively loaded into sEVs. (b) Coverage of MS2-gRNA. NGS samples were prepared from a plasmid library consisting of 13,145 MS2-gRNAs (see Methods for construction) or RNAs extracted from sEVs released from cells expressing CD63-MCP and transduced with the library. Read counts for every single gRNA were divided by the total count of the sample, then multiplied by the number of gRNAs in the library (13,145) to calculate normalized read count so that the mean value is 1. The barcoding efficacy was not as high as with the CD63-dCas9-based system.

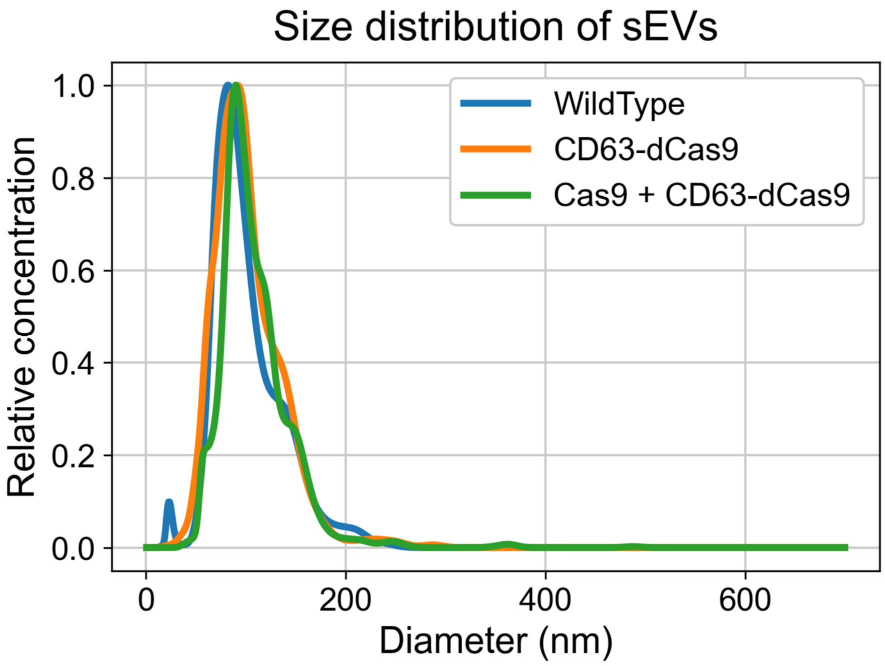

Supplementary Figure 4 | Results of nanoparticle tracking analysis (NTA) for sEVs isolated from culture media (CM) of HEK293T cells expressing each component. The concentration and size distribution of sEVs were measured with a NanoSight LM10. The lines show averaged concentration from 3 sequential measurements of each sample.

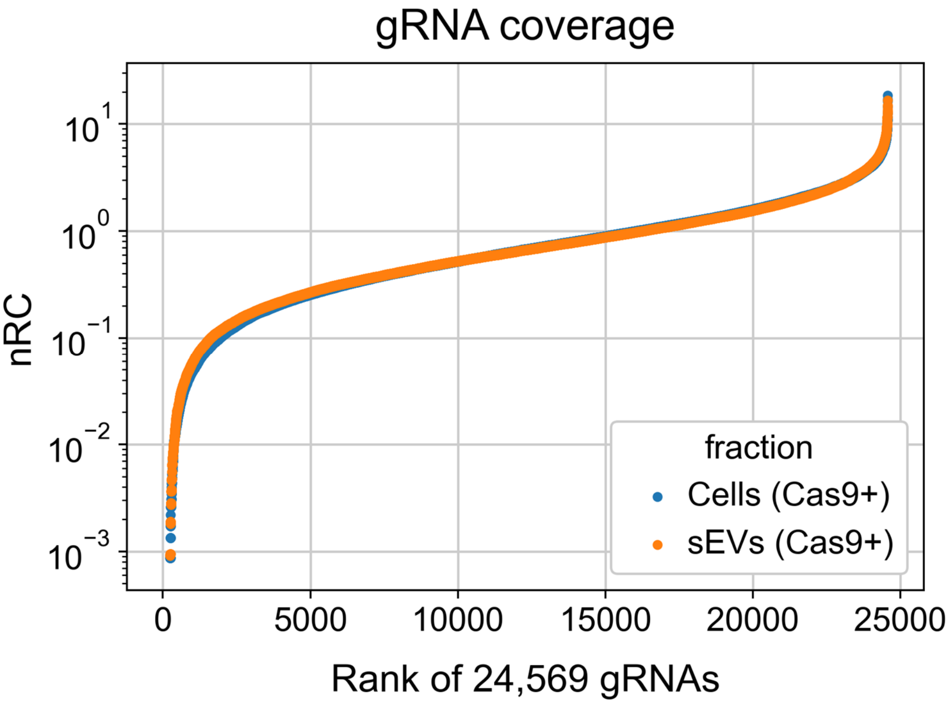

Supplementary Figure 5 | Confirmation that co-expression of Cas9 has little effect on coverage of gRNAs in sEVs. Cells expressing Cas9 and CD63-dCas9 were transduced with a library of 24,569 gRNAs. RNAs extracted from cells and sEVs released from them were processed for NGS read-out.

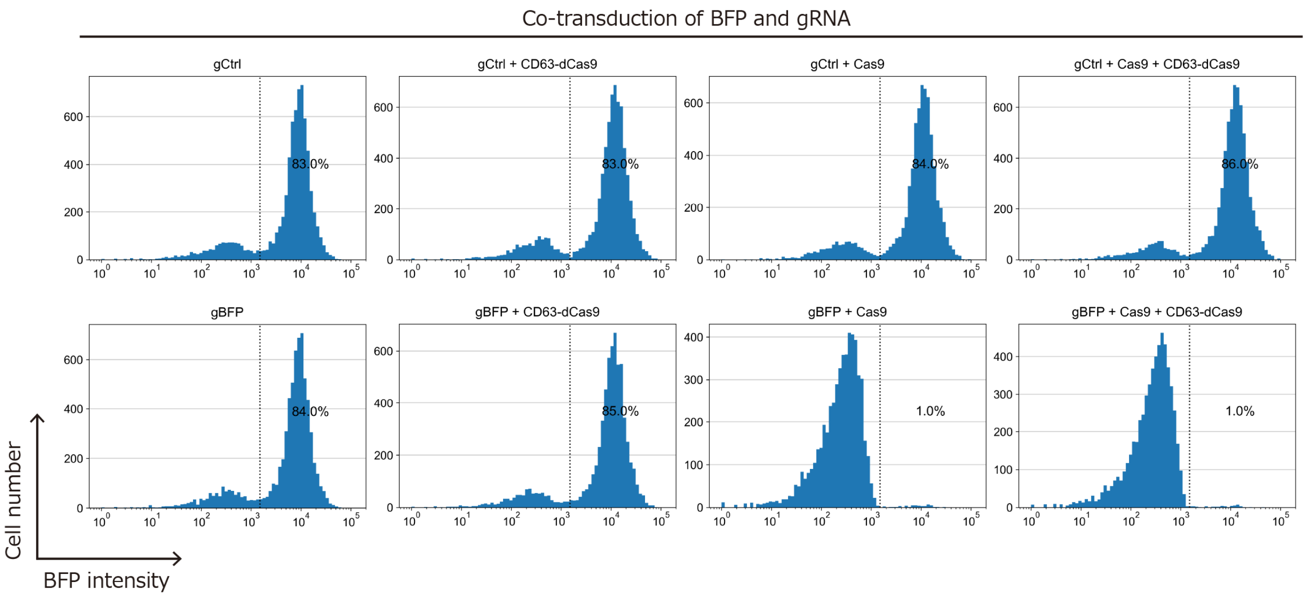

Supplementary Figure 6 | Confirmation that CD63-dCas9 expression has little effect on Cas9-induced gene KO. Flow cytometry analyses of HEK293T cells transduced with BFP and either control gRNA (gCtrl, pKK90) or BFP-targeting gRNA (gBFP, pKK209) are shown. BFP-derived fluorescence disappeared in cells expressing Cas9 and gBFP regardless of the expression of CD63-dCas9. The values in the plots show the proportion of BFP^{+} cells.

(a)

cat. #	symbol	Description	Genes targeted	gRNAs
101926	ACOC	Apoptosis and cancer	3,015	31,324
101927	DTKP	Drug targets, kinases, phosphatases	2,333	24,569
101930	PROT	Proteostasis	2,927	30,403
101931	TMMO	Trafficking, mitochondrial, motility	2,252	23,615

(b)

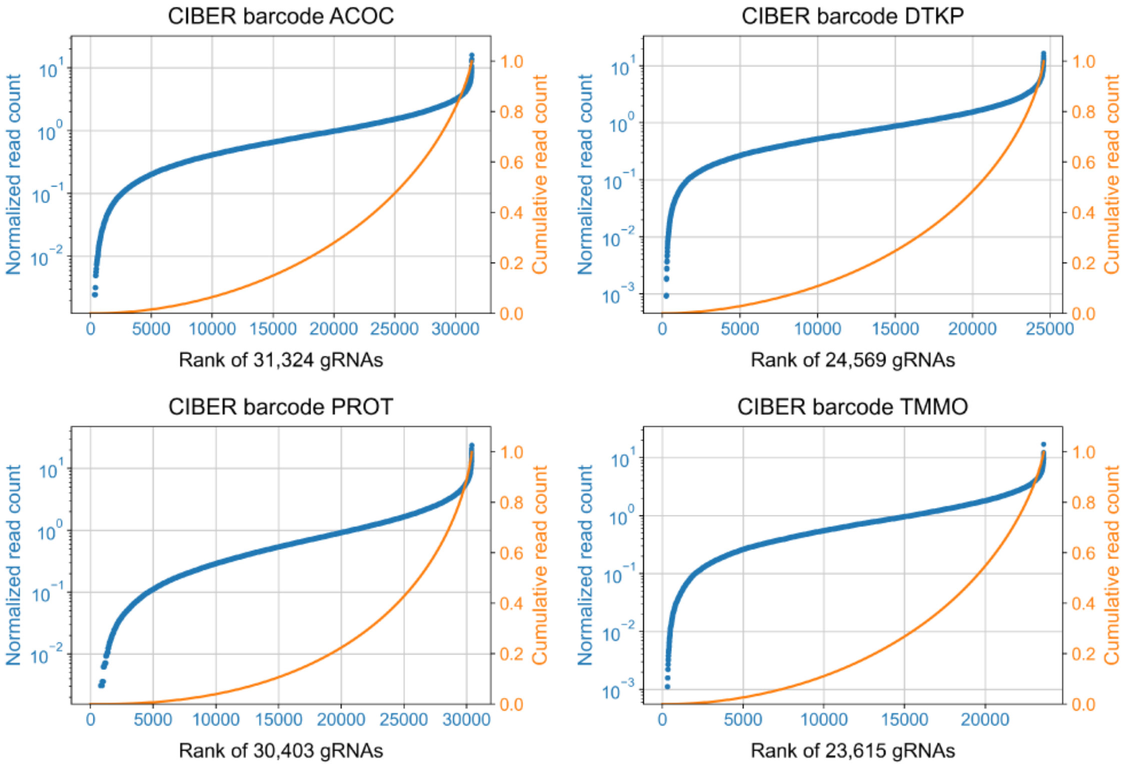

Supplementary Figure 7 | gRNA libraries used in this study and their barcoding performance in sEVs. (a) The four listed sub-pool libraries of Bassik deletion libraries[8] covering about half the genome in total were used. The spacer sequences are available in Supplementary data 7. (b) Data showing the high sEV barcoding efficacy using each gRNA library with HEK293T cells expressing CD63-dCas9 and Cas9 (the blue line obtained with the DTKP library is the same as the orange line in Supplementary Fig. 5).

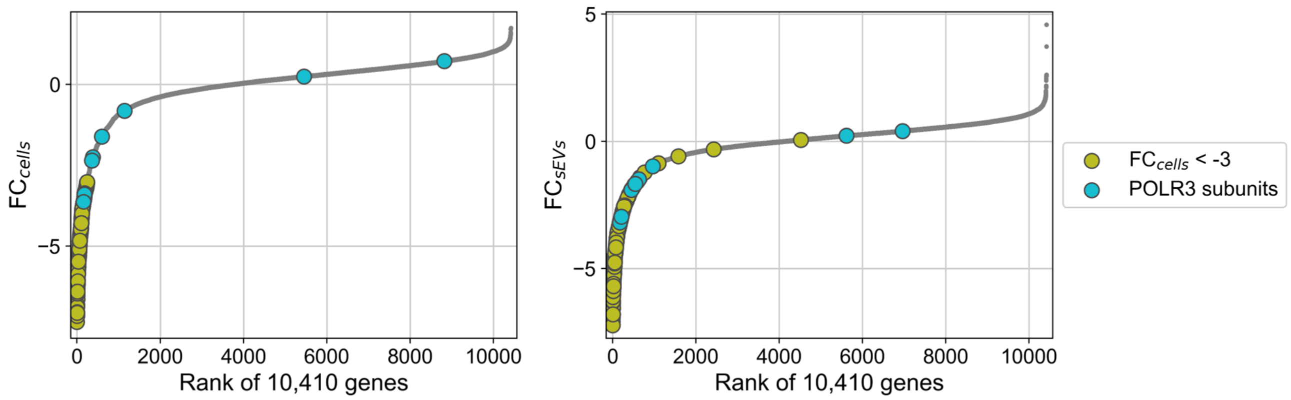

Supplementary Figure 8 | Evaluation of the effect of cellular gRNA level on gRNA amount in sEVs. The median value of z-normalized $\log_2$ (fold-change) of barcode gRNAs grouped by target gene in cells (FC_{cells}) and sEVs (FC_{sEVs}) are shown. gRNAs targeting POLR3 subunits (regulating gRNA transcription) as well as gRNAs targeting genes showing FC_{cells} less than -3 are highlighted. The results indicate that the sEV gRNA level is highly dependent on the cellular gRNA level, showing the necessity of taking this into consideration for extracting true sEV release regulators.

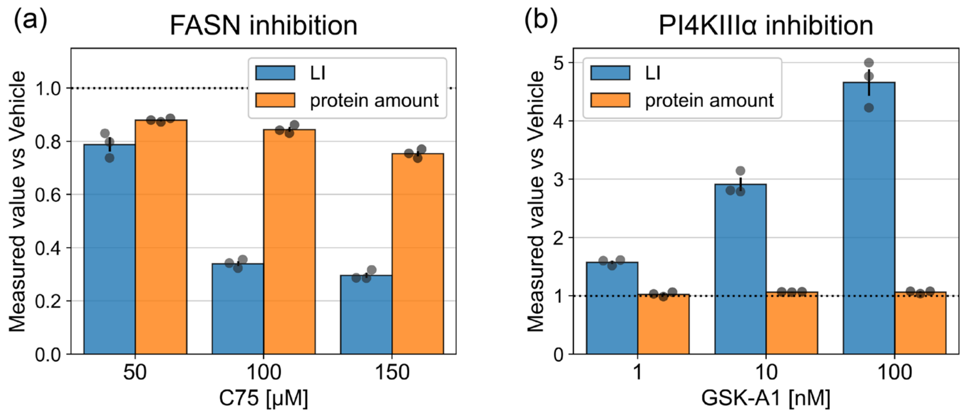

Supplementary Figure 9 | Raw measurement of LI and protein amount used to generate Fig. 3b and c. Measured LI (reflecting the sEV amount in the supernatant) and protein amount in the cellular fraction (measured by BCA assay, reflecting the cellular viability) are shown as relative values to the Vehicle condition. LIs are divided by the corresponding normalized protein amount to calculate the Normalized LI shown in Fig. 3b and c. Error bars represent ± SEM of biological replicates (n = 3).

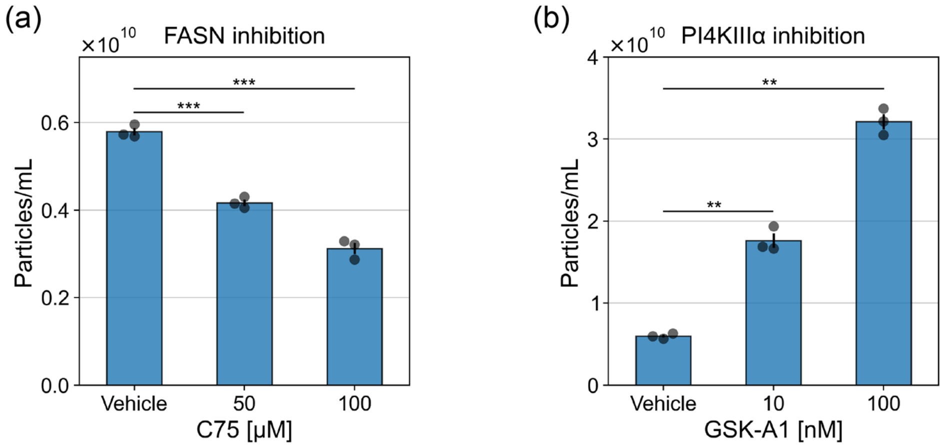

Supplementary Figure 10 | Concentration of sEVs released from HEK293T cells treated with C75 (FASN inhibitor) GSK-A1 (PI4KA inhibitor). NTA histograms in Fig. 3d and e were converted to bar graphs showing the particle numbers. Error bars represent ± SEM of biological replicates (n = 3). p: two-tailed Welch's t-test with Holm correction. $^{**}p < 0.005$, $^{***}p < 0.0005$

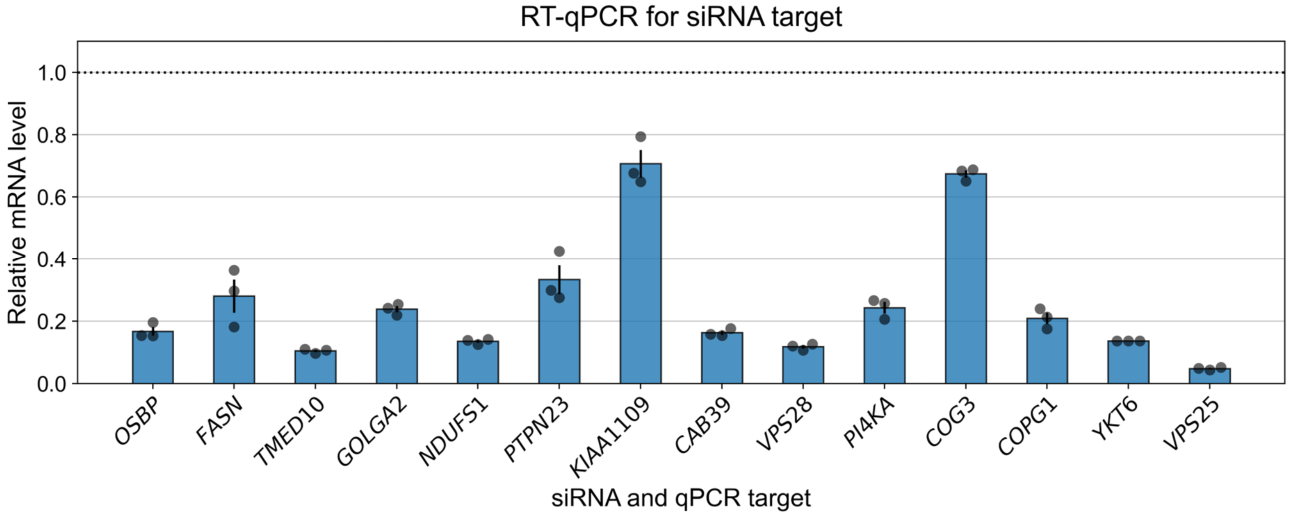

Supplementary Figure 11 | Confirmation of gene knockdown by siRNA used for validation in Fig. 3f. Cells were transfected with siRNA targeting one of the hit genes and incubated for 24 hours before lysis for qPCR quantification. Relative mRNA level was calculated using the $2^{-\Delta\Delta Ct}$ method with GAPDH as an internal control[13]. Error bars represent ± SEM of biological replicates (n = 3).

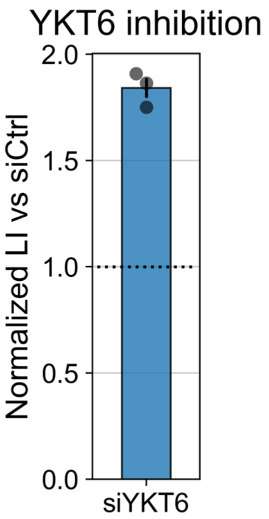

Supplementary Figure 12 | CD63-nluc reporter assay for YKT6 (z-RE = -3.40) by siRNAs. Protocol of the assay was the same as Fig. 3f. $p = 0.011$ by two-tailed Welch's t-test with Holm correction (with 14 samples).

Note regarding Supplementary Figure 12 and main Figure 3

For the orthogonal validation assay, we arbitrary selected genes to test taking the following points into consideration; 1. z-REs in gene level have large absolute values 2. REs in gRNA level consistently showed a similar tendency. Overall, 10 genes out of 14 genes were judged as true hits, which we believe sufficiently supports the usefulness of CIBER screening. Genes not validated were *YKT6* (z-RE = -3.40), *COG3* (z-RE = -3.20), *COPG1* (z-RE = -5.00), and *VPS25* (z-RE = 4.28). Knockdown of *COG3, COPG1*, and *VPS25* showed a change of the nluc signal as predicted by the CIBER screening in one experiment, but this was not reproducible, so further validation will be needed. Therefore, we did not count these genes as validated hits. As for YKT6, while this gene was detected as a lower hit in the CIBER screening, knockdown of this gene consistently upregulated the release of $CD63^+$ sEVs. It should be noted that Lu et al obtained completely opposite results regarding *YKT6* in their screening (bEXOMiR-based screening)[14]; in their screening, KO of *YKT6* was predicted to enhance sEV release, but their validation result suggested that the KO of *YKT6* suppressed the sEV release (their validation matches the result of CIBER screening, though their screening predicted the opposite effect). Additionally, there are two other controversial reports about whether *YKT6* enhances or suppresses sEV biogenesis[15,16]. These conflicting observations suggest that the function of *YKT6* may be sensitive to the experimental setting, as discussed in the case of the ESCRT proteins. Thus, it might be worth checking the function of the genes if a gene has z-RE of high absolute values, regardless of plus or minus).

Rank	Gene	Rank	Gene	Rank	Gene	Rank	Gene	Rank	Gene	Rank	Gene	Rank	Gene	Rank	Gene	Rank	Gene	Rank	Gene
1	CD9	101	ATP6V1A	201	CLIC4	301	H2AFX	401	PCBP2	501	DLG1	601	SPTBN1	701	CORO1C	801	HIST1H2AG	901	SERPING1
2	ACTB	102	PRDX2	202	EEF1G	302	RPLP2	402	WARS	502	RPS25	602	RPL28	702	CLDN4	802	HIST1H2AI	902	TOM1L2
3	CD81	103	ACLY	203	TXN	303	SERPINA1	403	HIST2H2BF	503	RPL18	603	LYPLA1	703	HIST1H2AD	803	KRT12	903	SYPL1
4	HSPA8	104	PROM1	204	CCT3	304	XRCC5	404	ATP6V1E1	504	RPL11	604	HIST1H1D	704	LYPLA2	804	VARS	904	CORO1B
5	CD63	105	UBA1	205	PSMA5	305	JUP	405	SLC12A1	505	PTPRJ	605	RPL35A	705	ATP5A1	805	PSMC6	905	ACE2
6	GAPDH	106	BROX	206	RRAS	306	HIST1H2BG	406	RAB21	506	RPL10A	606	GPRC5C	706	PPP2R1B	806	ALDH9A1	906	TAOK1
7	PKM	107	YWHAH	207	SLC16A1	307	PDIA3	407	PTGR1	507	PCNA	607	EPS8L1	707	PSMD5	807	RHOB	907	PTP4A1
8	ANXA2	108	RAN	208	SLC1A5	308	POTEF	408	AP2A1	508	ACOT7	608	HIST1H2BN	708	TXNRD1	808	AP2M1	908	TWF2
9	SDCBP	109	TUBA1B	209	HSP90B1	309	MYL12B	409	IMPDH2	509	KRT6A	609	LTF	709	RAB22A	809	RHEB	909	RACGAP1
10	HSP90AA1	110	EPCAM	210	ARF4	310	GANAB	410	SLC1A4	510	MYO1B	610	PACSIN3	710	ATP1A2	810	FARSA	910	UPF1
11	ANXA5	111	ACTN1	211	PDCD10	311	YES1	411	ABHD14B	511	FKBP4	611	RAB15	711	HIST1H2BA	811	QDPR	911	ERP44
12	LDHA	112	FLOT2	212	RPS16	312	MYO1D	412	EIF5A	512	PLXNB2	612	PSMD13	712	C11orf54	812	HPGD	912	CYBRD1
13	HSPA1A	113	PSMA7	213	HIST1H2AE	313	RHOG	413	HINT1	513	DARS	613	DPP3	713	PTPRF	813	KIF23	913	HNRNPU
14	YWHAE	114	RAB14	214	AKR1A1	314	ALDOC	414	HRAS	514	NAMPT	614	KIF5B	714	EIF2S1	814	GSS	914	EIF5
15	YWHAZ	115	RAB5A	215	ACTBL2	315	AARS	415	RPS24	515	CA2	615	RPL27	715	ATP1A3	815	GSTM2	915	CAV1
16	PDCD6IP	116	CAND1	216	ARPC2	316	RAB13	416	DDT	516	EIF4A2	616	RPL23	716	PABPC1	816	EPHB3	916	RAB25
17	ENO1	117	CCT5	217	ITGA3	317	ACTA2	417	GPRC5A	517	GFPT1	617	TMPRSS2	717	NAPG	817	HIST1H2AA	917	UBE2K
18	ALB	118	TAGLN2	218	LSR	318	NT5E	418	RPL4	518	VPS35	618	UMOD	718	RAB18	818	KRT3	918	COL18A1
19	MSN	119	GNAI3	219	RPS8	319	HIST1H2BL	419	HIST1H2BF	519	KRT6C	619	NAP1L1	719	CRK	819	7-Sep	919	HCK
20	EEF1A1	120	RPS27A	220	PGD	320	CYFIP1	420	SCARB2	520	HPRT1	620	NACA	720	EFNB1	820	DOPEY2	920	USP9X
21	CFL1	121	PSMA4	221	KRT2	321	SOD1	421	PLS3	521	VAMP8	621	PEPD	721	FABP5	821	SLK	921	TGM2
22	TPI1	122	CBR1	222	PSMB2	322	CAPZB	422	HEBP1	522	NPEPPS	622	APOA1	722	ATP4A	822	IGF2R	922	FAS
23	LDHB	123	CCT6A	223	TF	323	RRAS2	423	GGT1	523	EPRS	623	HIST1H2BJ	723	XPO1	823	CD55	923	COPS8
24	ACTN4	124	MUC1	224	HSPA6	324	F11R	424	HIST2H2AC	524	DAK	624	VAMP3	724	GLG1	824	CEACAM5	924	RPS15
25	PFN1	125	ACTA1	225	PPP1CA	325	S100A11	425	HIST1H2BE	525	EIF3M	625	ACO1	725	CDK1	825	ENPEP	925	NANS
26	FASN	126	MDH1	226	HSPG2	326	HIST1H2BB	426	PDXK	526	KIF12	626	SARS	726	NCKAP1	826	UBC	926	CHP1
27	ANXA6	127	RAP2B	227	SNAP23	327	CHMP1B	427	CTNNB1	527	NCL	627	SELENBP1	727	KIAA0368	827	CS	927	RTN4
28	YWHAQ	128	A2M	228	SRC	328	RPS2	428	XRCC6	528	PSMD2	628	HSPH1	728	MYH11	828	PCBD1	928	KIF3A
29	GNAS	129	KRT1	229	RPS3	329	C3	429	S100A8	529	MARCKSL1	629	NUTF2	729	RPS19	829	GLIPR2	929	QPRT
30	PGK1	130	HLA-A	230	CSTB	330	CIB1	430	S100A9	530	ACY1	630	PSMB8	730	RAB3A	830	GPC1	930	ADSS
31	HSPA1B	131	PRDX5	231	PRSS8	331	B2M	431	VPS25	531	EHD3	631	GNAO1	731	BHMT	831	CANX	931	DCTN2
32	ALDOA	132	FLNA	232	GNAQ	332	MVP	432	MYH14	532	CAPN2	632	EPHB1	732	CAPN7	832	SLC25A5	932	CSNK2B
33	HIST1H4I	133	ACTG1	233	VCL	333	ITGA2	433	P4HB	533	STXBP3	633	PAFAH1B2	733	ASAH1	833	MGAM	933	ECH1
34	FLOT1	134	TUBB4B	234	CAPZA2	334	RUVBL2	434	KRT15	534	PA2G4	634	CYFIP2	734	KRT4	834	UPK2	934	SSRP1
35	EZR	135	SLC9A3R1	235	PSMB1	335	TUBB4A	435	H2AFV	535	UBE2V1	635	PSMC4	735	LGALS1	835	VAMP7	935	LMNA
36	LGALS3BP	136	PSMA3	236	DPEP1	336	ICAM1	436	KRT5	536	PFKP	636	CHMP6	736	C4B	836	CA4	936	HUWE1
37	HSP90AB1	137	CPNE3	237	ACE	337	RPS13	437	STX4	537	VIM	637	LAMA5	737	GNG5	837	MAN1A1	937	TOP1
38	PPIA	138	ANXA7	238	GNAI1	338	CAPNS1	438	KRT17	538	GART	638	TTN	738	RPL34	838	SERPINA3	938	ARPC5
39	ANXA1	139	SLC12A2	239	KRT19	339	VAT1	439	CLTCL1	539	HIST1H2BM	639	AK1	739	FRK	839	ACP2	939	EIF3F
40	TUBB	140	PSMA6	240	KRT13	340	ASS1	440	PLEC	540	CAD	640	PAFAH1B1	740	GIPC1	840	ACTG2	940	RPL22
41	RAB5C	141	VPS37B	241	UBB	341	KRT16	441	HIST1H2BC	541	HYOU1	641	SYNCRIP	741	ACTR1B	841	VPS26A	941	GNPNAT1
42	TSG101	142	TUBA1A	242	RHOC	342	CTNND1	442	DNAJA2	542	PYGL	642	LYZ	742	FBP1	842	MPST	942	SGTA
43	PRDX1	143	CALM2	243	CALM1	343	ENO3	443	IPO5	543	RNPEP	643	LYN	743	PHB	843	TNPO1	943	CSNK2A1
44	CLTC	144	CNDP2	244	ANPEP	344	MYH10	444	FSCN1	544	PSMC5	644	HIST1H2AH	744	EIF4A3	844	KARS	944	ANKFY1
45	RAP1B	145	MFGE8	245	PGLS	345	SLC7A5	445	DDAH1	545	USP7	645	CUL4A	745	H2AFY	845	PRKCSH	945	RPL37A
46	GNB2	146	TUBA4A	246	CCT8	346	KRAS	446	PLS1	546	PRKAR2A	646	HNRNPH1	746	PGM1	846	AP1G1	946	ERP29
47	CLIC1	147	UBE2N	247	CALM3	347	DNAJA1	447	RPS15A	547	UBR4	647	KNG1	747	TUBA3C	847	ANP32A	947	FH
48	GPI	148	TKT	248	PIGR	348	APEH	448	RAC3	548	SET	648	GPRC5B	748	MST4	848	CHMP1A	948	DDX6
49	EEF2	149	NRAS	249	ENO2	349	NME2	449	EPHA2	549	QSOX1	649	SLC16A3	749	HIST1H2AK	849	PSMD1	949	PSMB7
50	YWHAG	150	ACTR3	250	MARCKS	350	MDH2	450	PSAT1	550	NQO1	650	AHNAK	750	MPP5	850	PCMT1	950	UBE2M
51	RAB7A	151	LAMP1	251	MYL6	351	GRB2	451	RPL30	551	RPS10	651	DERA	751	ITIH4	851	PSMC2	951	CSNK1A1
52	RDX	152	KPNB1	252	RPS5	352	CAB39	452	TNIK	552	TPP2	652	AP1B1	752	PTK7	852	FKBP1A	952	HGS
53	GNB1	153	ARHGDIA	253	APOE	353	CSE1L	453	CD46	553	RPL24	653	FDPS	753	PLOD3	853	RAD23B	953	ATP6V0D1
54	RAB10	154	TUBB3	254	APRT	354	CAPZA1	454	TUBB1	554	PYGB	654	TSPAN8	754	HPX	854	SEC24C	954	RBP5
55	YWHAB	155	PARK7	255	KRT8	355	PNP	455	AP2A2	555	GNB4	655	ARL3	755	GNPDA1	855	GNA14	955	LRP2
56	GNAI2	156	CHMP5	256	ANXA3	356	HNRNPK	456	TPM3	556	HSPA9	656	SDC4	756	PTP4A2	856	ARHGDIB	956	H3F3C
57	CDC42	157	PSMB3	257	KRT14	357	HIST2H2BF	457	BAIAP2L1	557	SDCBP2	657	FGF	757	PSME2	857	BLVRA	957	TBCA
58	VCP	158	KRT10	258	RNH1	358	GDI1	458	RPS11	558	HIST1H2AC	658	PSMD3	758	H2AFZ	858	PSMC1	958	PDZK1
59	RALA	159	CAP1	259	DYNC1H1	359	NAPA	459	STIP1	559	PACSIN2	659	GMDS	759	GGH	859	DNAJC13	959	DDB1
60	ATP1A1	160	ARPC4	260	UEVLD	360	STK24	460	CD2AP	560	PPP2CA	660	RAP2A	760	MYO6	860	ATP6V0A1	960	RPLP1
61	MYH9	161	TCP1	261	RPL12	361	HNRNPA2B1	461	AP2B1	561	KRT76	661	HBB	761	CUL4B	861	LIN7C	961	ILK
62	CLU	162	RAB2A	262	RPS18	362	PPA1	462	RAB43	562	NME1-NME2	662	TPP1	762	RHOF	862	AHCYL1	962	SLC25A3
63	PDCD6	163	EIF4A1	263	ATP5B	363	AKR1B1	463	PSMD7	563	KRT24	663	SERPINB6	763	CHMP3	863	C9	963	NOTCH1
64	HSPA5	164	TOLLIP	264	ST13	364	LAMC1	464	ESD	564	PRDX4	664	DDX3X	764	SNRPD3	864	PTPRA	964	DNPEP
65	AHCY	165	ACTR2	265	RAB6A	365	PFKL	465	EGFR	565	SCRIB	665	AK2	765	PRKACA	865	XPO7	965	FABP1
66	CHMP2A	166	PHGDH	266	PCBP1	366	PSMB4	466	VTN	566	CDH1	666	DDAH2	766	ITGB5	866	TUBA3E	966	TUFM
67	EHD4	167	PPIB	267	IDH1	367	RPS7	467	RPL7	567	MARS	667	RPL23A	767	PTGES3	867	PFAS	967	LIN7A
68	ARF1	168	CCT7	268	EPS8L2	368	RAB3D	468	RAB3B	568	DCD	668	COTL1	768	PGAM2	868	RPL14	968	FTH1
69	RAB1A	169	SLC2A1	269	DSP	369	ARPC3	469	ALDH1A1	569	SNRNP200	669	PARP1	769	GNAL	869	MYO1E	969	APCS
70	MYO1C	170	PGAM1	270	OLA1	370	TPM4	470	CLDN3	570	VDAC1	670	AKR7A2	770	CP	870	YBX1	970	SLC12A7
71	IST1	171	ARF6	271	ITGAV	371	ALDOB	471	THY1	571	PPP1CC	671	PSMC3	771	DNM2	871	ALDH7A1	971	COPG1
72	RAP1A	172	ARF5	272	FLNB	372	CRYZ	472	ITGB4	572	SLC29A1	672	GCN1L1	772	RPL31	872	EIF3K	972	PSMA8
73	HSPA2	173	FN1	273	TUBB2A	373	SORD	473	G6PD	573	ACPP	673	DCXR	773	HNRNPF	873	ABCC1	973	CKAP5
74	ANXA4	174	HIST1H2AB	274	ATIC	374	PSME1	474	GSTA1	574	SPR	674	RAB27A	774	CPM	874	ITGB3	974	UPK1A
75	MIF	175	HSPD1	275	UGP2	375	UGDH	475	HLA-B	575	RARS	675	FLNC	775	LCK	875	TUBA3D	975	SKP1
76	RAB35	176	PAICS	276	GSN	376	MME	476	ABCB1	576	HIST1H2BK	676	DDX39B	776	EFHD2	876	SYNGR2	976	EPHB4
77	GDI2	177	ADAM10	277	TUBB6	377	TARS	477	RPL13	577	PPP1CB	677	TUBB8	777	CST3	877	IPO7	977	RPL35
78	GSTP1	178	GOT1	278	ACTR1A	378	PDIA6	478	KRT18	578	STX3	678	ARPC1B	778	HIST2H2AB	878	TUBB2B	978	UBL3
79	DPP4	179	CD44	279	KRT6B	379	TGFBI	479	APP	579	DOCK9	679	EIF3A	779	PLSCR1	879	KRT27	979	FTL
80	RHOA	180	WDR1	280	GSTO1	380	THBS1	480	RUVBL1	580	DHX9	680	AHSG	780	FGG	880	ITCH	980	HSPE1
81	VPS28	181	MITD1	281	CFL2	381	HNRNPC	481	CKB	581	CMPK1	681	IARS	781	SERPINA5	881	PKP3	981	ITM2B
82	RAB8B	182	BSG	282	RPS14	382	CTSD	482	RPS20	582	GRHPR	682	RSU1	782	H3F3B	882	RPL13A	982	ERBB2
83	TFRC	183	KRT9	283	ITGA6	383	TLN1	483	PSMD6	583	EEF1E1	683	SCARB1	783	FGB	883	ACSL4	983	ATP2B2
84	HSPB1	184	PSMB6	284	CCT4	384	VASP	484	LCP1	584	SLC12A3	684	NAPSA	784	RPS28	884	NEDD4L	984	CALB1
85	RAB5B	185	RALB	285	RPS4X	385	DSTN	485	PSAP	585	IQGAP2	685	RPL19	785	PRKDC	885	USP14	985	MYL6B
86	RAB1B	186	RAC2	286	RPL3	386	NCSTN	486	RPL15	586	MASP2	686	SNRPD2	786	PSMD12	886	PDIA4	986	PPL
87	PRDX6	187	TUBA1C	287	SLC44A1	387	ATP6V1B1	487	EPHB2	587	LTA4H	687	NARS	787	LMAN2	887	CARS	987	PPP1R7
88	ACTC1	188	RAB11B	288	KRT7	388	CTNNA1	488	MINK1	588	CPNE1	688	RP2	788	EIF2S3	888	PLCB3	988	MTAP
89	ANXA11	189	HLA-DRA	289	HSPA4	389	RPL7A	489	RAB27B	589	STX7	689	LASP1	789	DBI	889	RPS12	989	SERPINF1
90	SFN	190	GNA11	290	LAP3	390	MTHFD1	490	HNRNPD	590	SND1	690	CAPN5	790	OLFM4	890	ATP6V1H	990	S100P
91	EHD1	191	LAMP2	291	PSMB5	391	AQP2	491	RPL5	591	EIF3B	691	H3F3A	791	TPM1	891	GBF1	991	GAA
92	CCT2	192	EEF1A2	292	VPS4B	392	XPNPEP2	492	HIST1H2BH	592	RPS6	692	OTUB1	792	ABCE1	892	SERINC1	992	PKLR
93	PEBP1	193	PTGFRN	293	NPM1	393	ACAT2	493	MAP4K4	593	PTBP1	693	PICALM	793	ETF1	893	TUBA8	993	MPO
94	HSPA1L	194	PSMA1	294	RAB11A	394	ATP6V1B2	494	GARS	594	SPTAN1	694	ARPC5L	794	BANF1	894	CDC37	994	ADSL
95	RAC1	195	SRI	295	EPS8	395	GSTM3	495	CALR	595	PPP2R1A	695	DLD	795	ROCK2	895	ALCAM	995	MAT2A
96	ITGB1	196	STOM	296	CAPN1	396	TALDO1	496	2-Sep	596	ACP1	696	STXBP2	796	LARS	896	LAMB1	996	NUMA1
97	IQGAP1	197	RPSA	297	S100A6	397	RPS9	497	UBA52	597	RPL8	697	RPL17	797	CPNE8	897	C4A	997	MPP6
98	CHMP4B	198	RPLP0	298	GNB2L1	398	GSR	498	GOLGA7	598	GLO1	698	APOD	798	FGA	898	CACYBP	998	TRIP10
99	RAB8A	199	LGALS3	299	CHMP2B	399	RPS3A	499	VIL1	599	PSMD11	699	TJP2	799	TINAGL1	899	RPN2	999	MARK2
100	GNA13	200	CNP	300	EPB41L2	400	DPYSL2	500	HRSP12	600	SDC1	700	FYN	800	GSTA2	900	ADH5	1000	SNX18

Rank
1-250
251-500
501-750
751-1000

Gene group
Lower hits
N.S.
Upper hits

Supplementary Figure 13 | Matching of the hit genes of CD63-CIBER and top 1,000 sEVs-associated proteins compiled from the VesiclePedia database[17,18] (related to Fig. 3g). The lower/upper hits in CD63-CIBER screen are colored blue/orange, respectively.

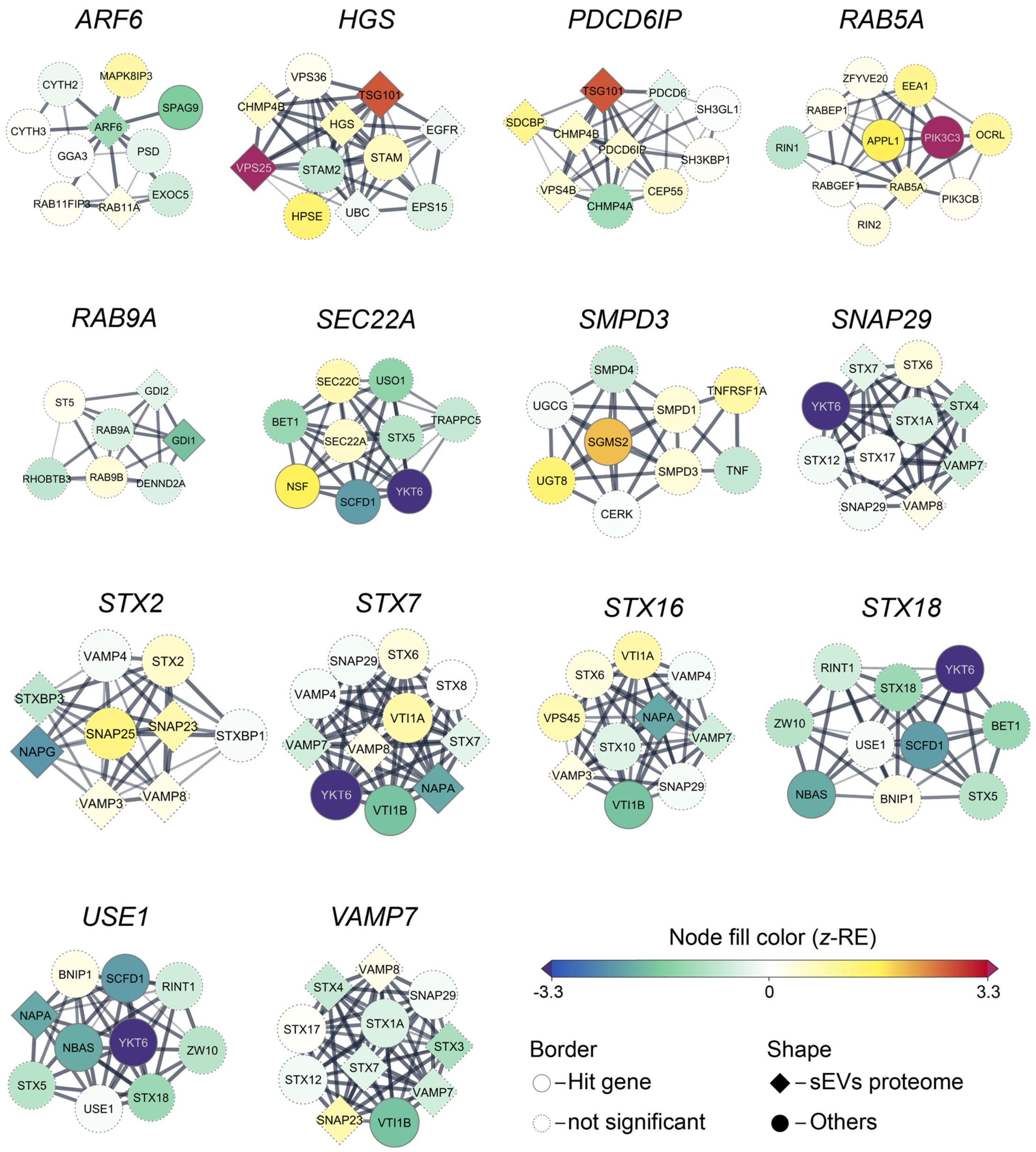

Supplementary Figure 14 | Connection of the hit genes in CD63-CIBER with known sEV release regulators (*ARF6, HGS, PDCD6IP, RAB5A, RAB9A, SEC22A, SMPD3, SNAP29, STX2, STX7, STX16, STX18, USE1* and *VAMP7*) analyzed by STRING. For each known regulator, the interaction network with functionally connected proteins is displayed under the gene names. Each node represents a gene product, and the node color corresponds to the *z*-RE. Hit genes have a rigid line edge.

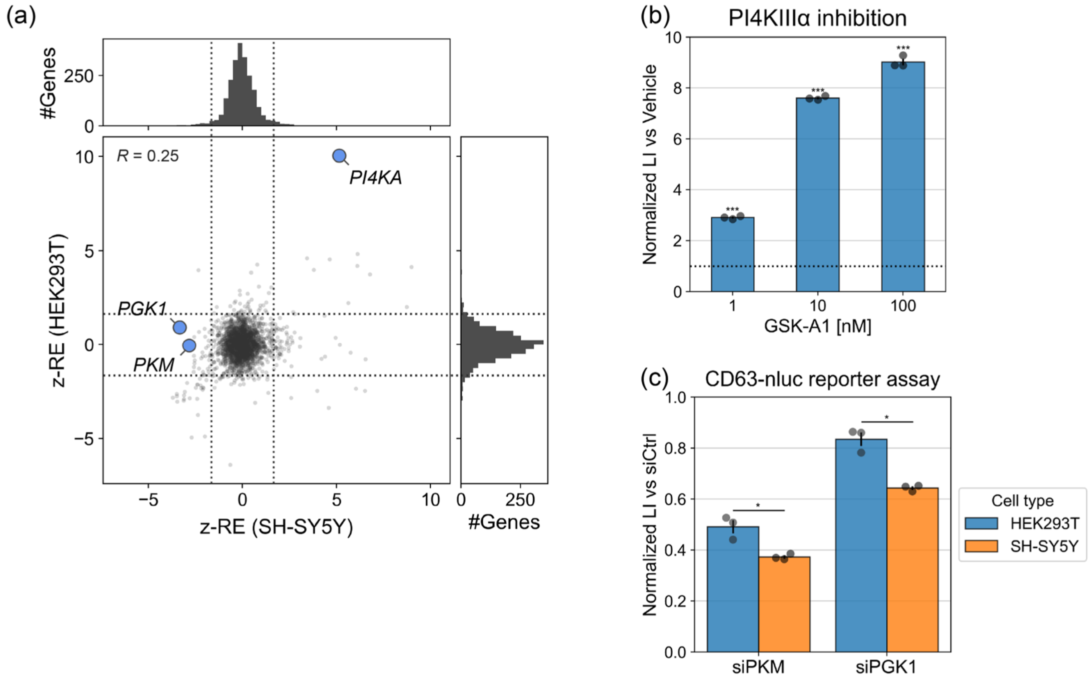

Supplementary Figure 15 | Results of small-scale CD63-CIBER screening (using the DTKP library) with SH-SY5Y cells and comparison with the results for HEK293T cells. (a) The pair-wise plot of *z*-REs obtained from CD63-CIBER screening in HEK293T and SH-SY5Y performed with the DTKP library targeting 2,333 genes (Supplementary Fig. 7a). Dashed line shows *z*-RE of +/- 1.65. (b) Validation of the effects of *PI4KA* by CD63-nluc reporter assay. SH-SY5Y cells stably expressing CD63-nluc were treated with various concentrations of GSK-A1 and the amount of sEVs released from the cells was estimated by luminescence signal as was done in Fig. 3c. (c) Validation of the effect of *PKM* and *PGK1* by siRNA with CD63-nluc reporter assay. Error bars represent ± SEM of biological replicates (n = 3). p: two-tailed Welch's t-test with Holm correction. *p < 0.05, **p < 0.005, ***p < 0.0005.

Note regarding Supplementary Figure 15

We conducted a small-scale CD63-CIBER screening with the DTKP library (2,333 genes with 24,569 gRNAs, drug targets, kinases, and phosphatases) in SH-SY5Y cells (neuroblastoma derived). This screening was successful, supporting the portability of CIBER screening to other cell lines. *PI4KA* was again detected as one of the top upper hits and validated to be a true hit, suggesting that this gene works as a potent sEV release regulator in various cell lines (note that many hit genes in HEK293T cells such as *CD63* and *FASN* are not included in the DTKP library). We also tried to find cancer-specific lower hits. We focused on *PKM* and *PGK1* as candidate SH-SY5Y-specific lower hits. The knockdown of these genes indeed decreased the CD63^{+} sEV release more significantly in SH-SY5Y cells than in HEK293T cells, showing the potential of CIBER screening to find cell-type-specific sEV release regulators. It should be noted that the cell-type specificity was not perfect, because moderate suppression of CD63^{+} sEV release was also observed in HEK293T cells. Genome-wide screening with a greater number of cell lines with robust bioinformatic analysis might be beneficial to find genes whose KD/KO ratios show large differences among different cell types.

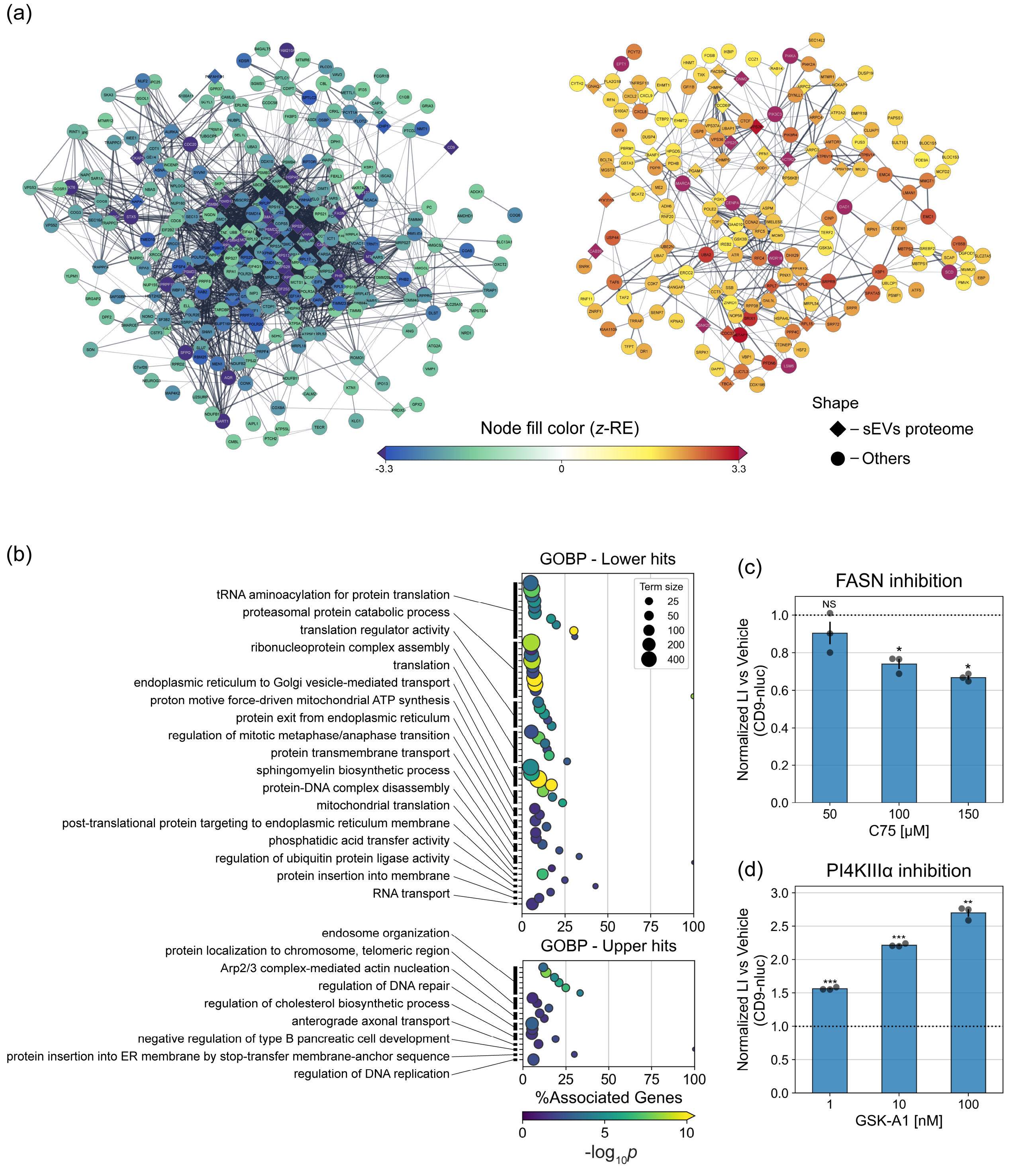

Supplementary Figure 16 | Results of CD9-CIBER screening. (a) STRING analysis with lower hits and upper hits of CD9-CIBER screening. Each node represents a gene product, and the node color corresponds to the *z*-RE. Only nodes connected to at least one other node are shown. The nodes of proteins found in the sEVs proteome (Supplementary Fig. 13) are shown as diamond shapes while others are shown as circles. (b) The results of GOBP enrichment analysis. The *x*-axis represents the gene ratio, which refers to the ratio of lower- or upper-hit genes to all gene numbers annotated to the term. The circle size indicates the total number of genes annotated to the term. The circle color indicates the -log$_{10}$(p-value) by two-tailed Fisher's exact test with Holm

correction. Only terms with adjusted p-value lower than 0.05 are displayed. Terms are grouped based on their kappa score level (0.4) calculated using ClueGO app and represented by the most significant term in each group. (c, d) Validation of the effect of *PI4KA* and *FASN* with the CD9-nanoluc (nluc) reporter assay. HEK293T cells stably expressing CD9-nluc were treated as described in Fig. 3b and c. Error bars represent ± SEM of biological replicates (n = 3). p: two-tailed Welch's t-test with Holm correction. *$p < 0.05$, **$p < 0.005$, ***$p < 0.0005$, NS, not significant.

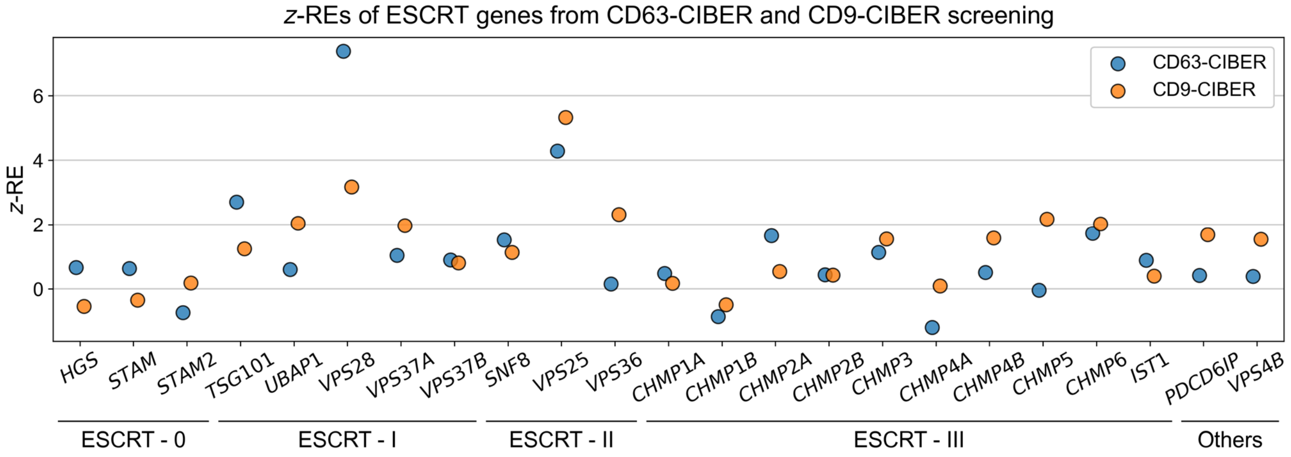

Supplementary Figure 17 | z-REs of ESCRT genes obtained from CD63- and CD9-CIBER screening. $R = 0.63$.

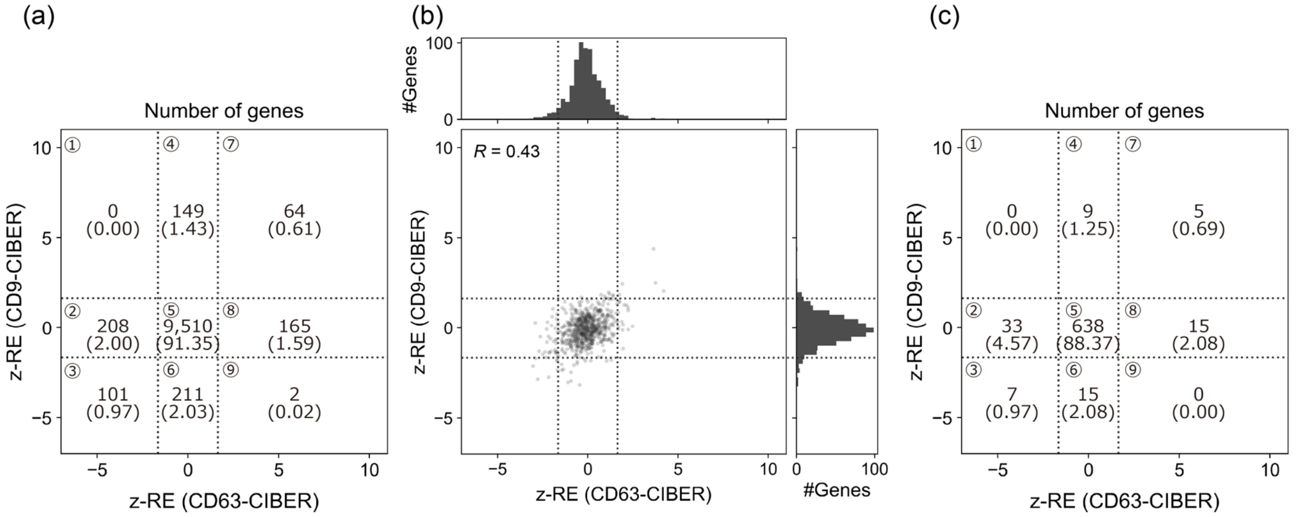

(d)

Term	Term size	CD63-CIBER		CD9-CIBER	
		#Associated genes	adjp	#Associated genes	adjp
NADH dehydrogenase (ubiquinone) activity	42	11	1.14.E-08	2	0.654
mitochondrial electron transport, cytochrome c to oxygen	24	6	3.01.E-04	1	0.683
mitochondrial electron transport, NADH to ubiquinone	53	11	1.69.E-07	2	0.697
proton motive force-driven mitochondrial ATP synthesis	68	14	1.02.E-09	7	0.015
oxidoreduction-driven active transmembrane transporter activity	78	16	3.33.E-11	4	0.780
proton-transporting ATP synthase activity, rotational mechanism	20	4	3.09.E-02	3	0.280
aerobic electron transport chain	96	19	3.64.E-13	4	1.000
mitochondrial ATP synthesis coupled electron transport	104	19	1.71.E-12	4	0.844
oxidative phosphorylation	150	24	1.22.E-14	9	0.086
ATP biosynthetic process	107	15	5.53.E-08	8	0.043
aerobic respiration	202	27	1.50.E-14	10	0.189
mitochondrial respiratory chain complex assembly	96	11	9.11.E-05	6	0.350
nucleoside triphosphate biosynthetic process	140	16	3.04.E-07	8	0.204
primary active transmembrane transporter activity	195	18	9.07.E-07	5	0.395
purine ribonucleoside triphosphate metabolic process	260	21	5.59.E-07	11	0.379
nucleoside triphosphate metabolic process	286	22	5.80.E-07	11	0.853
proton transmembrane transport	170	11	1.67.E-02	6	1.000
nucleotide biosynthetic process	335	21	1.64.E-04	10	0.940
ribonucleotide metabolic process	519	26	1.22.E-04	14	0.868

(e)

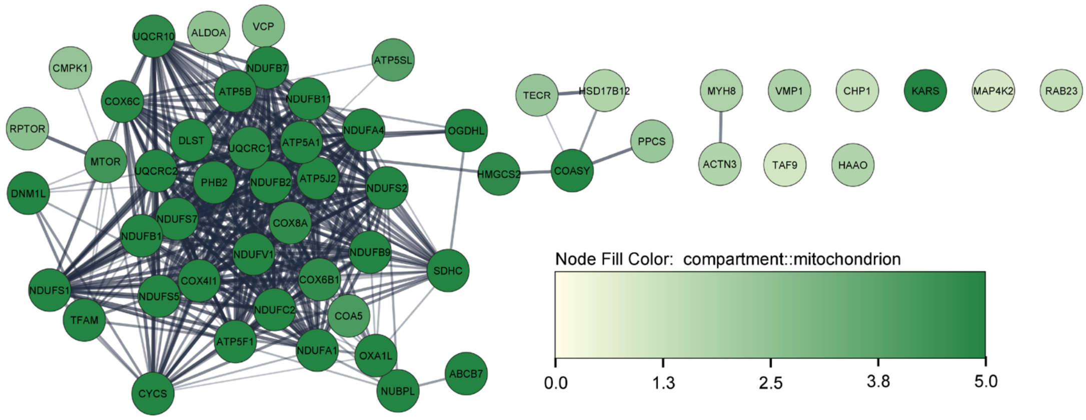

Supplementary Figure 18 | Detailed analysis of OxPhos terms and their annotated genes. (a) The number of genes in each region of Fig. 4a separated by -1.65 and 1.65 of *z*-REs for both *x*- and *y*-axis. Percentages are shown in parentheses underneath each gene number. (b) Pair-wise plot of *z*-REs obtained from CD63/CD9-CIBER screening for 722 genes annotated to OxPhos terms. Dashed lines show +/-1.65. (c) The number of

genes in each region of Supplementary Fig. 18b. OxPhos genes are concentrated to CD63-CIBER-specific lower hits (middle left region; region 2) compared to all the screened genes (p = 0.00012: Fisher's exact test). (d) Table of OxPhos terms significant for lower hits of CD63-CIBER. "Term size" shows the number of genes annotated to a term in each row. "#Associated genes" shows the number of genes annotated to the term and detected as hits in each screening. adjp; p values by Fisher's exact test adjusted by Bonferroni step down. (e) STRING analysis of OxPhos genes on Fig. 4b (*z*-RE lower than -1.65 in either screen). Node color corresponds to mitochondrial associations according to Cytoscape. The density of the edge is proportional to the strength of PPI.

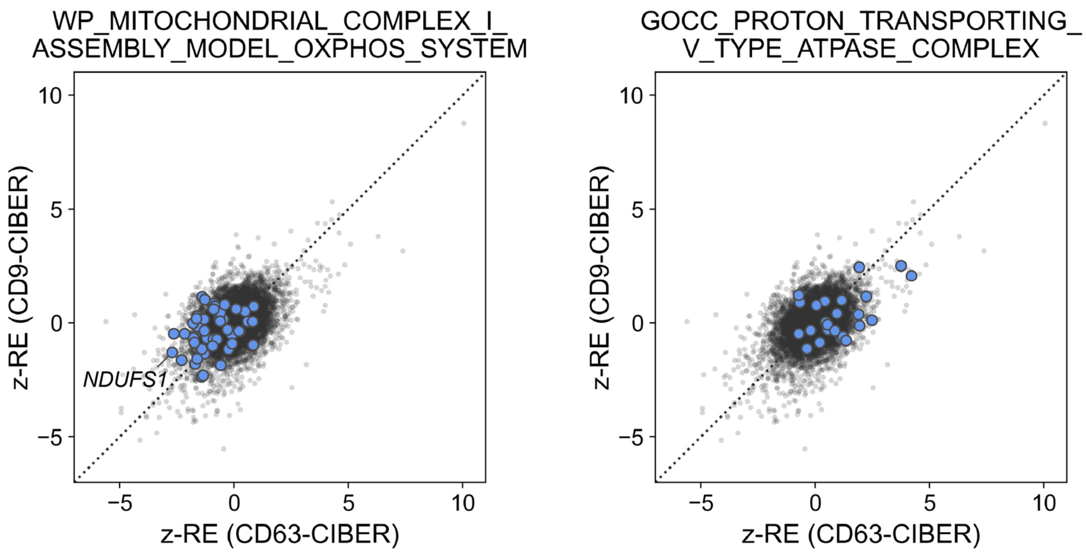

Supplementary Figure 19 | Highlighted scatter plot of genes annotated to either of the GO terms in Fig. 4c, overlaid on the comparative scatter plot of Fig. 4a with a dashed diagonal line. Along with the GSEAPreranked results, in CD63-CIBER compared to CD9-CIBER, more than half of genes (32/49) annotated to WP_MITOCHONDRIAL_COMPLEX_I_ASSEMBLY_MODEL_OXPHOS_SYSTEM have lower *z*-REs, and 19 out of 25 genes annotated to GOCC_PROTON_TRANSPORTING_V_TYPE_ATPASE_COMPLEX have higher *z*-REs.

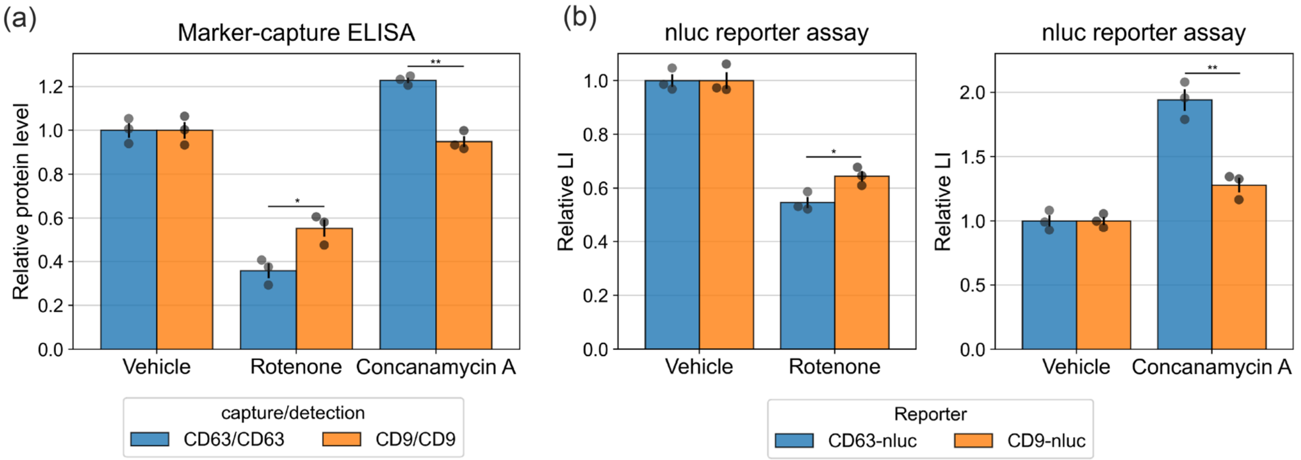

Supplementary Figure 20 | Orthogonal validation of the effect of rotenone and concanamycin A. (a) Wild-type HEK293T cells were treated with rotenone at 10 nM or concanamycin A at 1 nM for 24 hours. The amounts of CD63$^+$ and CD9$^+$ sEVs in culture supernatant were separately measured by sandwich ELISA. (b) HEK293T cells stably expressing CD63-nluc or CD9-nluc were treated with rotenone at 3 nM or concanamycin A at 0.3 nM. The amounts of CD63$^+$ and CD9$^+$ sEVs in culture supernatant were measured in terms of nluc intensity. Error bars represent ± SEM of biological replicates (n = 3). p: two-tailed Welch's t-test with Holm correction for (a). *$p < 0.05$, **$p < 0.005$.

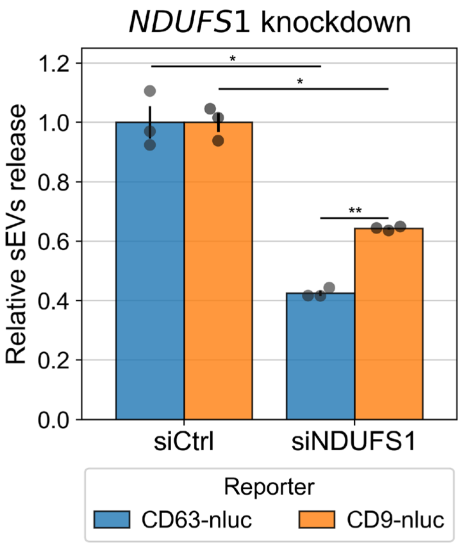

Supplementary Figure 21 | CD63-nluc and CD9-nluc reporter assays for *NDUFS1* (z-RE = -2.71 in CD63-CIBER, -1.29 in CD9-CIBER) using siRNAs. Assay protocol was the same as in Fig. 3f. The data of CD63-nluc cells are derived from Fig. 3f. p: two-tailed Welch's t-test with Holm correction. *$p < 0.05$, **$p < 0.005$.

Note related to Supplementary Figure 20, 21 and main Figure 4d

We conducted validation regarding the stronger effect of mitochondrial function on the release of CD63$^+$ sEVs compared to CD9$^+$ sEVs with three different methods: PS-capture ELISA (Fig. 4d), EV-marker-sandwich ELISA (CD63-CD63 or CD9-CD9, Supplementary Fig. 20a) and nluc-reporter assay (Supplementary Fig. 20b). All the results are consistent and confirmed that the release of CD63$^+$ sEVs was reduced more significantly than that of CD9$^+$ sEVs, though the release of CD9$^+$ sEVs was also reduced to some extent. This is in line with our GSEAPreranked result showing that the gene set WP_MITOCHONDRIAL_COMPLEX_I_ASSEMBLY_MODEL_OXPHOS_SYSTEM has a significant negative enrichment score and that knockdown of *NDUFS1*, one of the major mitochondrial complex I subunits, reduced the release of not only CD63$^+$ sEVs, but also CD9$^+$ sEVs to some extent (Supplementary Fig. 21), as predicted by the result of CIBER screening (*z*-RE = -2.71 in CD63-CIBER, -1.29 in CD9-CIBER). Note that blocking mitochondrial function affects various biological pathways including the cell cycle (e.g., rotenone is also known to be a cell cycle inhibitor[19]), and we have shown that halting the cell cycle reduces the release of CD9$^+$ sEVs (Fig. 5b). So, it is natural that the release of CD9$^+$ sEVs is also affected by rotenone from this viewpoint. Still, we would like to emphasize that what we wanted to show was that we could identify pathways that act significantly differently on the release of different subpopulations of sEVs, and this purpose was achieved.

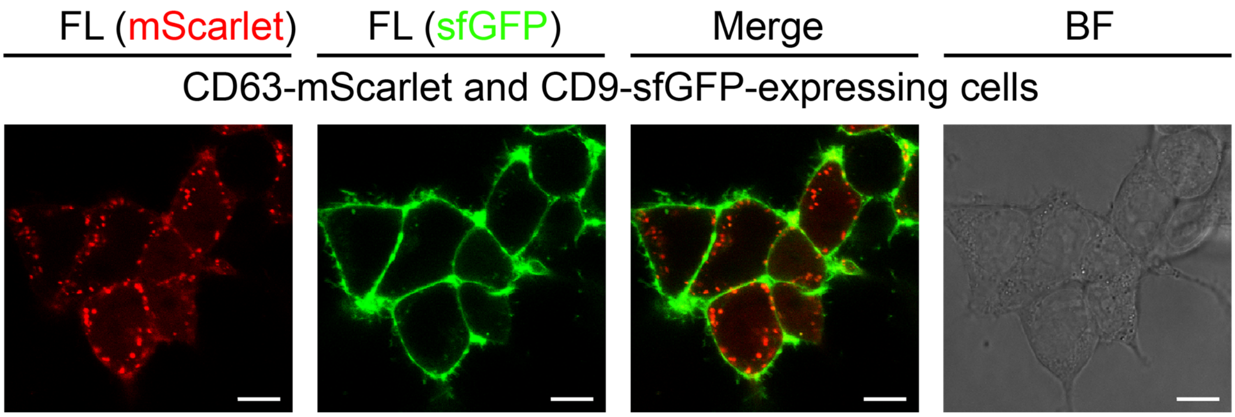

Supplementary Figure 22 | Subcellular localization study of CD63 and CD9. HEK293T cells seeded on an 8-well chamber were transiently co-transfected with CD9-sfGFP and CD63-mScarlet expression vectors. After a day of incubation, cells were visualized with a Leica SP8. Fluorescence images were captured with excitation and emission wavelengths of 488/510-555 nm for sfGFP and 569/579-665 nm for mScarlet. Scale bar, 10 µm.

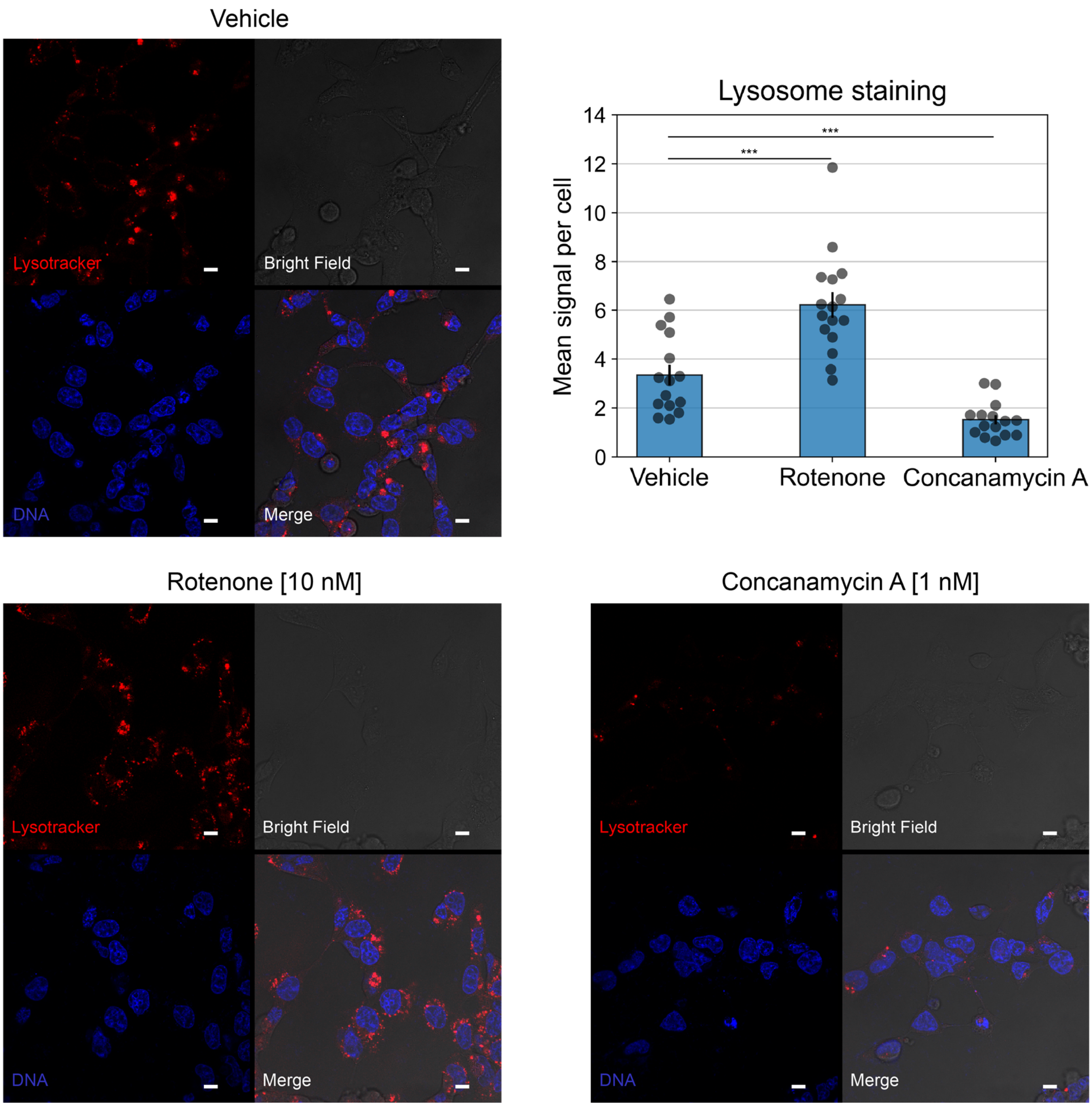

Supplementary Figure 23 | Confirmation of lysosomal activity upon treatment with inhibitors. HEK293T cells seeded on an 8-well chamber were treated with rotenone at 10 nM or concanamycin A at 1 nM for 24 hours. After the treatment, cells were stained with Hoechst33342 and then LysoTracker™ Red DND-99. Cells were visualized with a Leica SP8. Fluorescence images were captured with excitation and emission wavelengths of 577/584-718 nm for LysoTracker™ Red DND-99 and 405/440-510 nm for Hoechst33342. Scale bar, 10 µm. The boxplot at the upper left shows the mean LysoTracker™ signal per cell in the displayed image for each condition. Error bars represent ± SEM of fluorescence signal from 15~16 different cells in a single experiment. *p*: two-tailed Welch's *t*-test with Holm correction. ***$p < 0.0005$.

.

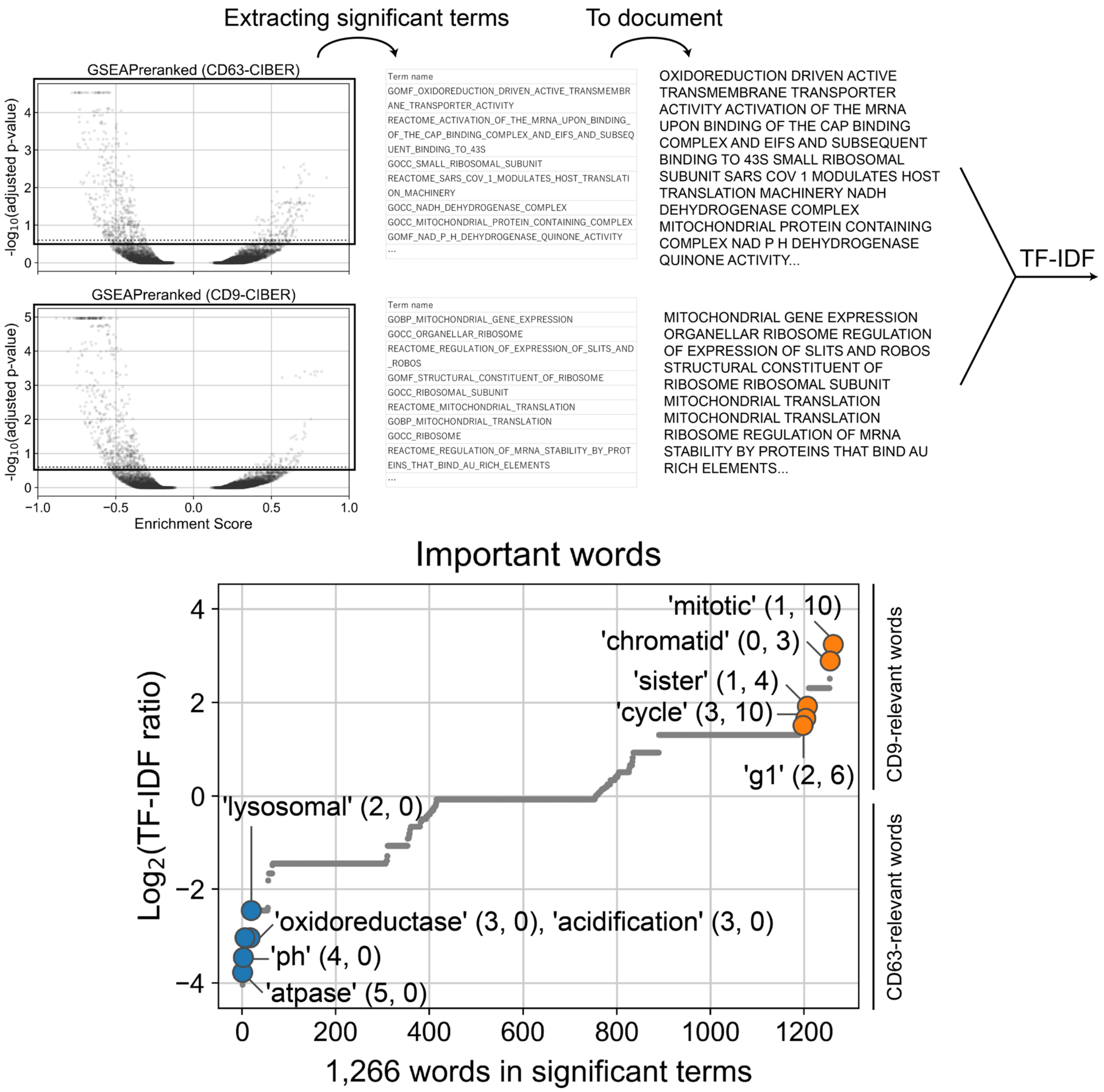

Supplementary Figure 24 | Term frequency - inverse document frequency (TF-IDF) analysis of hit terms. Words to be analyzed were collected from terms with adjusted *p*-value lower than 0.25 in the results of GSEAPreranked of CD63-CIBER and CD9-CIBER screening. The figure shows the $\log_2$ ratio of TF-IDF scores (CD9 vs CD63) from 1,266 words detected. Annotations were expressed as ‘word’ (count in CD63-CIBER, count in CD9-CIBER). This analysis suggests that the mitotic events are more relevant to $CD9^+$ sEVs (‘sister’ is used as SISTER_CHROMATID or SISTER_CHROMATIDS in terms, and 8 out of 10 ‘cycle’ were used as CELL_CYCLE in terms). Note that the $CD63^+$ EV-specific involvement in lysosomal activity is again detected in this analysis. See Supplementary Data 8 for individual TF-IDF scores.

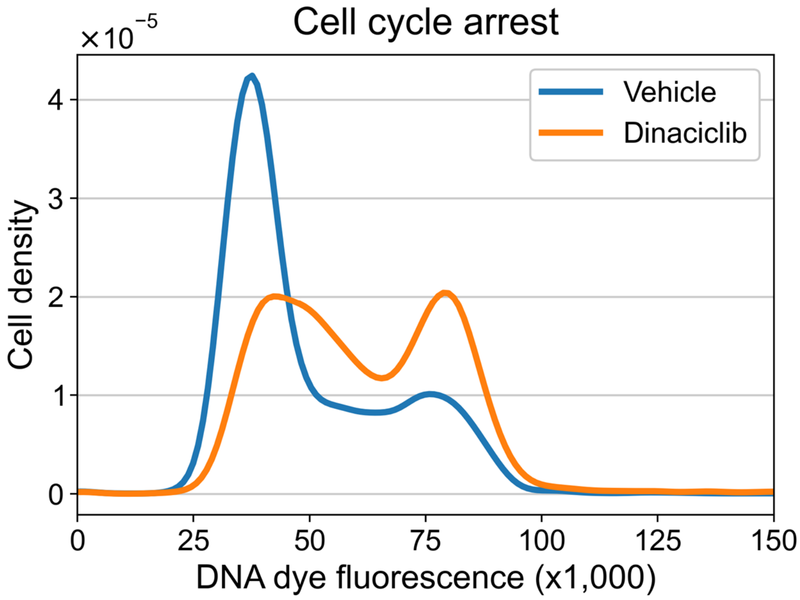

Supplementary Figure 25 | Flow cytometry analysis of cell cycle arrest by dinaciclib. HEK293T cells were treated with dinaciclib at 10 nM for 24 hours, stained with a DNA dye and analyzed by FCM. Curves for each condition shows kernel density estimation calculated from the FCM histogram. This data shows that the cells were successfully arrested at G2/M phase upon treatment with dinaciclib. $p < 10^{-16}$ by Kolmogorov Smirnov test.

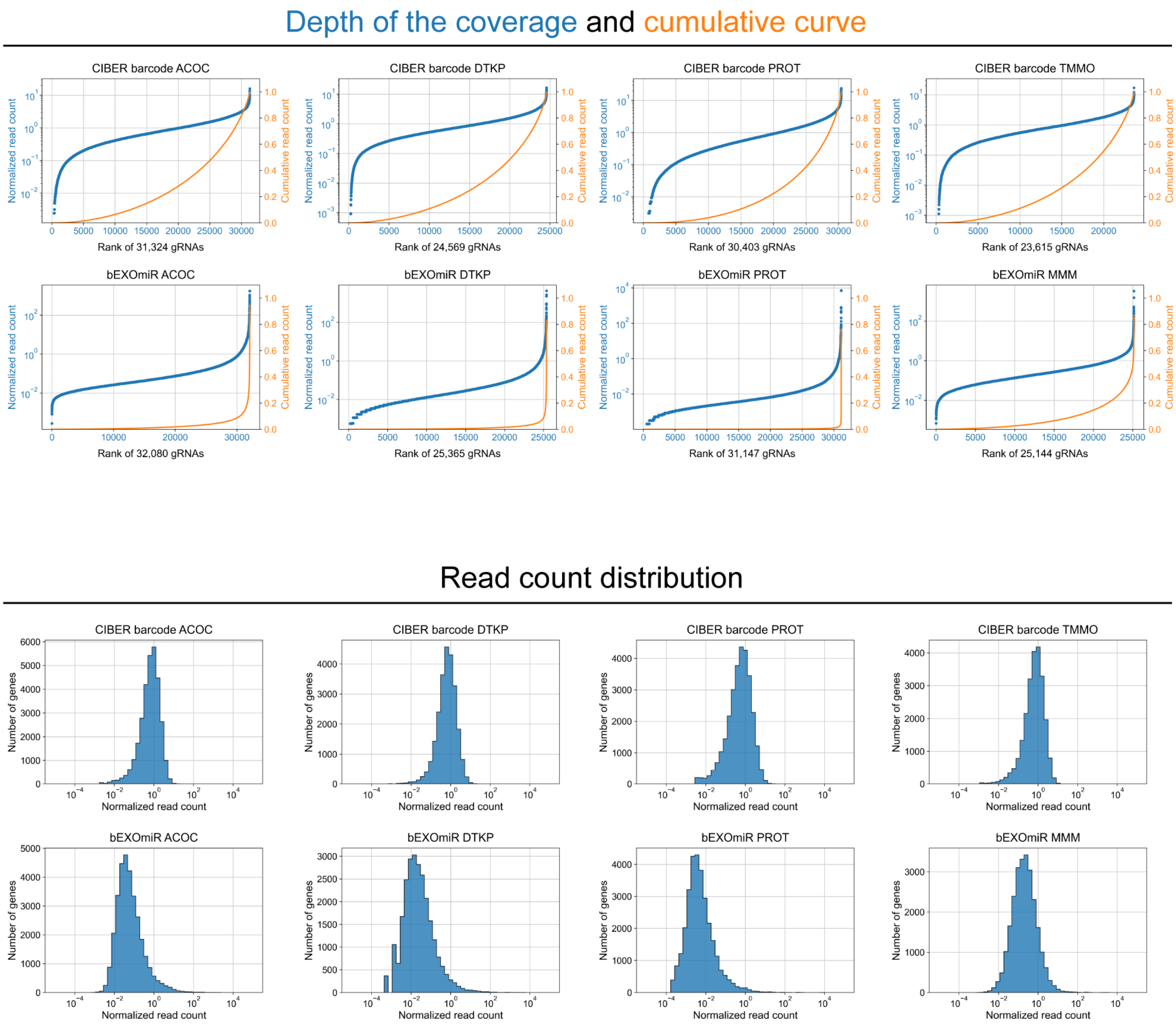

Supplementary Figure 26 | Comparison of sequencing results of CIBER screening and a previous study[14]. The upper panel shows the depth of barcode coverage from sEVs released by Cas9-expressing cells (the data of CIBER barcode ACOC/DTKP/PROT/TMMO is the same as shown in Supplementary Fig. 7b). In the CIBER-screening barcodes were uniformly detected, whereas most reads covered a limited population of barcodes in the previous report. The lower panel shows the histogram with the distribution of barcodes by read count. Barcodes are symmetrically distributed in CIBER screening while they are right-skewed in the previous report. These data support the superior reliability of sEV barcoding in the CIBER screening platform.

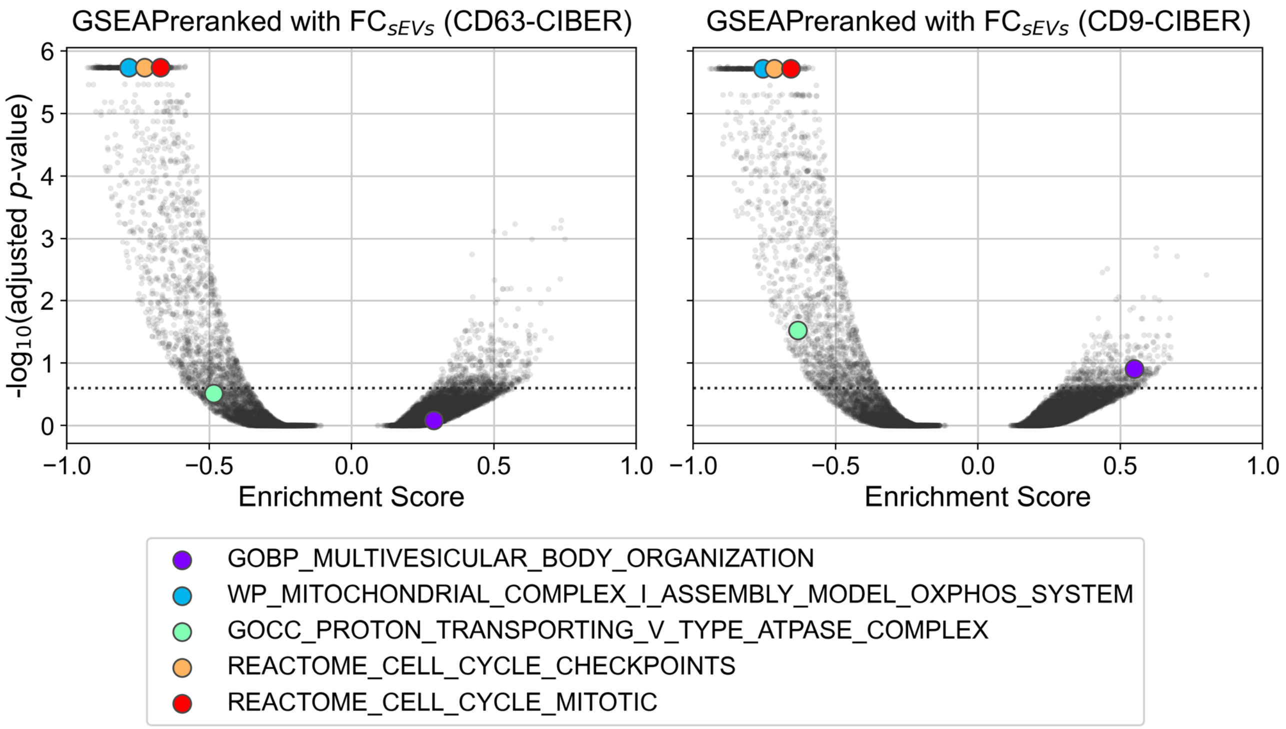

Supplementary Figure 27 | Volcano plot of enrichment score calculated via GSEAPreranked with z-FC$_{sEVs}$ as the query array. The dashed line shows adjusted *p*-value of 0.25 calculated by two-sided Kolmogorov Smirnov test with Benjamini Hochberg correction.. Because FC$_{sEVs}$ are highly correlated to FC$_{cells}$ (Fig. 2b), genes affecting cellular activity (e.g. viability) are often detected as false-positive lower hits in the screening when only FC$_{sEVs}$ are used to find hit genes. It is also impossible to see the different effect of the cell cycle on the release of CD63$^+$ sEVs and CD9$^+$ sEVs when z-FC$_{sEVs}$ are used as a pre-ranked query array.

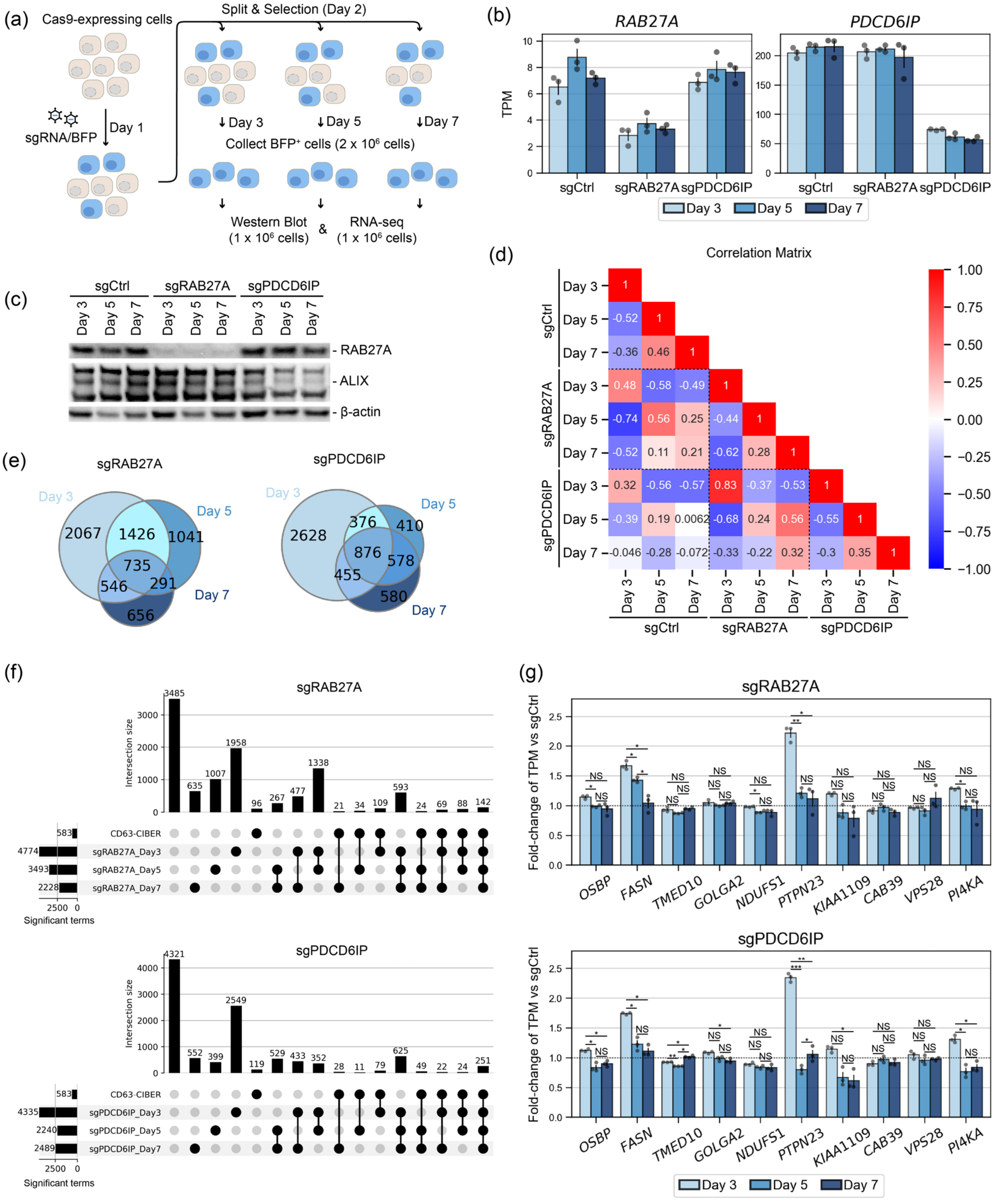

Supplementary Figure 28 | Time-dependent change of RNA expression profile after *RAB27A* and *PDCD6IP* KO in HEK293T cells. (a) Cas9-expressing HEK293T cells were transduced with lentivirus encoding sgRNA, BFP and puromycin resistance (pKK90 for sgCtrl, pKK275 for sgRAB27A, pKK278 for sgPDCD6IP) at Day 1. Transduced cells were selected with puromycin. At Day 3, 5 and 7, indicated number of BFP^{+} cells were collected by FACS and used for western blotting and RNA-sequencing. (b) Transcripts per million (TPM) of *RAB27A* and *PDCD6IP* of cells transduced with each sgRNA. (c) Western blot against RAB27A, ALIX (gene

product of PDCD6IP) and β-actin (control). (d) Correlation matrix representing Pearson's correlation coefficients of standardized TPM between all samples. TPM values for each transcript were scaled using *z*-standardization (mean of 0 and standard deviation of 1) across all samples to enable comparison. (e) Venn diagram showing the overlapped hit term. Fold-changes of TPM (sgRAB27A or sgPDCD6IP vs sgCtrl) for all the transcripts were calculated and queried for GSEAPreranked. The numbers of hit terms under the criterion of adjusted p-value < 0.25 in each region were displayed. (f) The UpSet plot visualizes the intersections of hit terms by GSEAPreranked in CD63-CIBER and each group in Fig. 28e, where each vertical bar represents the size of the intersection between specific groups. The horizontal bars indicate the total size of each individual group. The dots below the intersection bars represent the groups involved in each intersection, with connected dots indicating the specific combination of groups contributing to the intersection. The size of each intersection is shown on top of the bars. This plot highlights that for both *RAB27A* and *PDCD6IP* KO, hit terms in CD63-CIBER seem to be concentrated in gene sets that are consistently perturbed upon the KO of RAB27A/PDCD6IP, compared to the average of other irrelevant gene sets, suggesting that there may be some compensation related to sEV release process. (g) Fold-changes of TPM for experimentally validated hits of CD63-CIBER. p: two-tailed Welch's t-test with Holm correction within each transcript. *p < 0.05, **p < 0.005, ***p < 0.0005. NS, not significant.

Discussion regarding Supplementary Figure 28

We conducted experiments to examine the effect of the timeline of the experiments in order to address the issue of why some known sEV release regulators (e.g. RAB27A, PDCD6IP) were not detected in CIBER screening. For this purpose, we knocked out RAB27A and PDCD6IP with the gRNAs existing in the library used in our CIBER screening, and conducted RNA-seq at different time points after the transfection of gRNAs in HEK293T cells expressing Cas9 (Days 3, 5 and 7. Supplementary Fig. 28a, Supplementary Data 9). The reduction of the transcripts targeted by each gRNA through nonsense-mediated mRNA decay (NMD) was clearly observed in RNAseq results in all the samples, supporting the adequacy of genetic perturbation (Supplementary Fig. 28b). Western blotting shows complete removal of Rab27a at all time points, while expression of ALIX (gene product of PDCD6IP) was gradually reduced but still remained to some extent even at Day 7 (Supplementary Fig. 28c) (the half-life of ALIX in the cells is reported to be relatively long (37 hours)[20], so it is reasonable that some protein remained even at Day 7). This observation suggests that Day 7 (when we replaced culture media for sEVs collection) might be too early for analyzing the function of proteins with a long half-life, though analyzing at Day 7 is generally recommended for CRISPR KO screening[21]. The correlation coefficients of transcripts per million (TPM) were different when compared by time point (even without KO) (Supplementary Fig. 28d). This indicates that the results of assays are inherently affected by the difference of time points, making it difficult to directly compare the results with those of other studies. By GSEAPreranked analysis, we observed time-dependent changes in the expression of various gene sets upon KO of RAB27A and PDCD6IP (Supplementary Fig. 28e, Supplementary Data 6). Notably, hit gene sets of CIBER screening were actually more concentrated as gene sets whose expression was significantly and time-dependently changed, as

compared with non-CIBER-hit gene sets (Supplementary Fig. 28f). This suggests that the KO of the said genes affects the expression levels of other genes regulating sEV release, which may imply that some compensation related to sEV release occurred. Also, focusing on the individual CIBER-hit genes, the expression of multiple genes validated to affect sEV release, such as *PI4KA, FASN, YKT6, KIA1109*, and *PTPN23*, seemed to change depending on the assay time point, which further suggests that the assay timeline influences the results (Supplementary Fig. 28g).

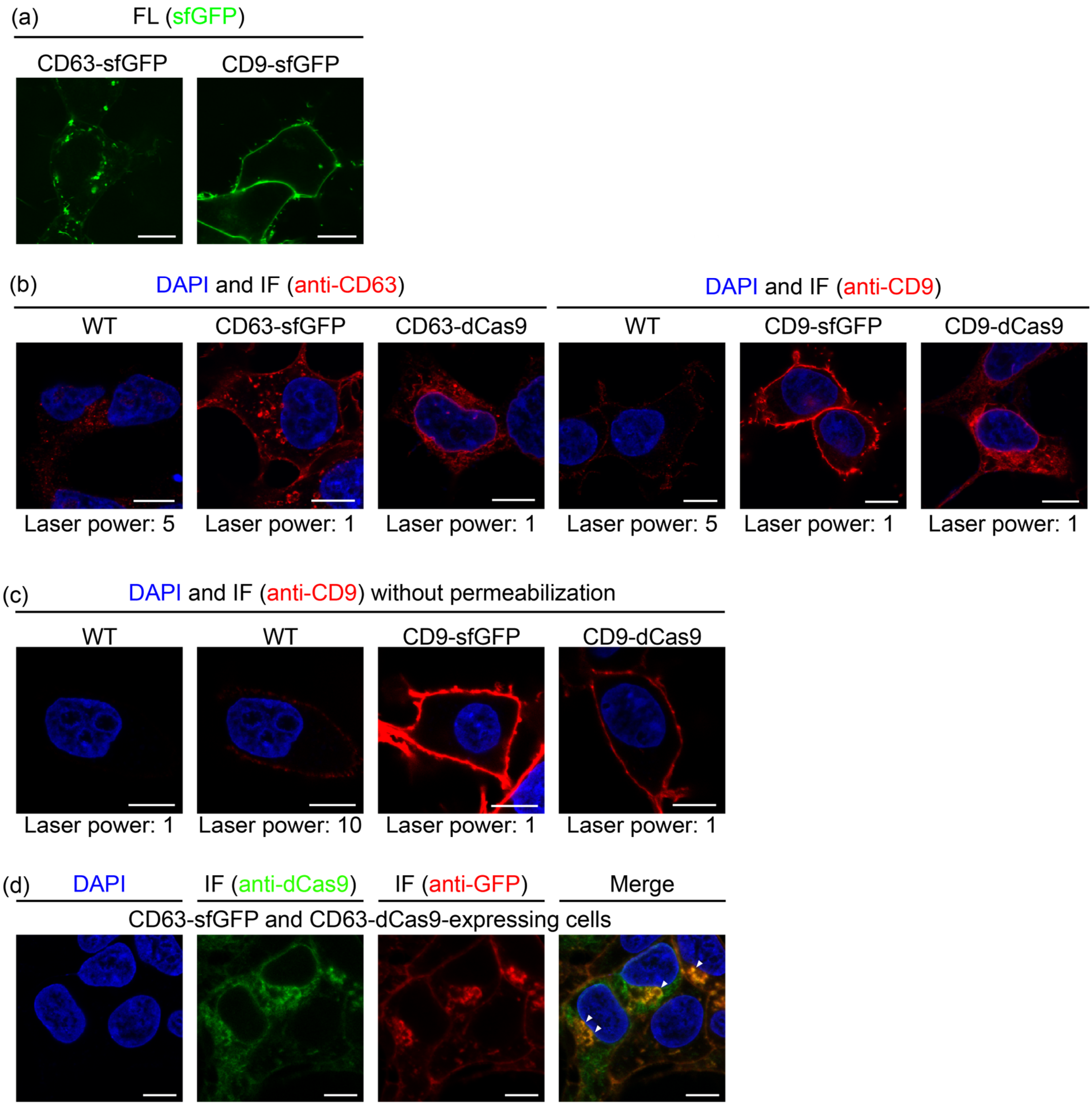

Supplementary Figure 29 | Subcellular localization study of CD63-dCas9 and CD9-dCas9 in HEK293T cells (compared with CD63/CD9-sfGFP). (a) Fluorescence images of live cells stably expressing CD63-sfGFP

(pKK150) or CD9-sfGFP (pKK151) (as references for immunofluorescence staining). Both components localize to their native destination: endosomes and plasma membrane, respectively. (b-d) Immunofluorescence images of wild-type (WT) cells and cells stably expressing exogenous components as displayed at the top of each image. (b) Immunofluorescence images of the cells stained with an anti-CD63 antibody or anti-CD9 antibody with permeabilization. Overexpressed CD63 and CD9 are so abundant that the laser power for excitation was 5 times less than that for visualizing native proteins. Localization of CD63-sfGFP and CD9-sfGFP is similar to the localization of those in live cell imaging (Supplementary Fig. 29a), suggesting that the localization of the protein can be properly monitored with immunofluorescence with our setting. With this setting, CD63-dCas9 and CD9-dCas9 showed ER-like inner-membrane-localized patterns, suggesting slow intracellular trafficking of the proteins. (c) Images of cells stained with an anti-CD9 antibody without permeabilization where only the extracellular epitopes were stained. The result supports the idea that CD9-dCas9 reaches the plasma membrane despite the ER-like staining pattern with permeabilization. (d) Images of CD63-sfGFP and CD63-dCas9-expressing cells stained with anti-GFP antibody and anti-dCas9-antibody. The white arrowheads in the merged image indicate the co-localization of CD63-dCas9 and CD63-sfGFP, which supports the view that CD63-dCas9 reaches the same destination as CD63-sfGFP that accumulates in the endosomal compartment, as is the case for native CD63 (see also supplementary Fig. 2 showing that CD63-dCas9 and CD9-dCas9 are indeed loaded in sEVs) (Note that pKK180 was used for the expression of CD63-dCas9, because pKK60 (used for the expression of CD63-dCas9 in other figures) co-expresses EGFP. See supplementary Table 1). Throughout the figure, the immunofluorescence signal shown in green is from an Alexa Fluor 488-conjugated secondary antibody, and the immunofluorescence signal shown in red is from an Alexa Fluor 594-conjugated secondary antibody. Images were captured with excitation and emission wavelengths of 405/440-480 nm for DAPI, 488/490-540 for Alexa Fluor 488-conjugated secondary antibody and sfGFP, and 559/565-620 nm for Alexa Fluor 594-conjugated secondary antibody. Scale bar, 10 µm.

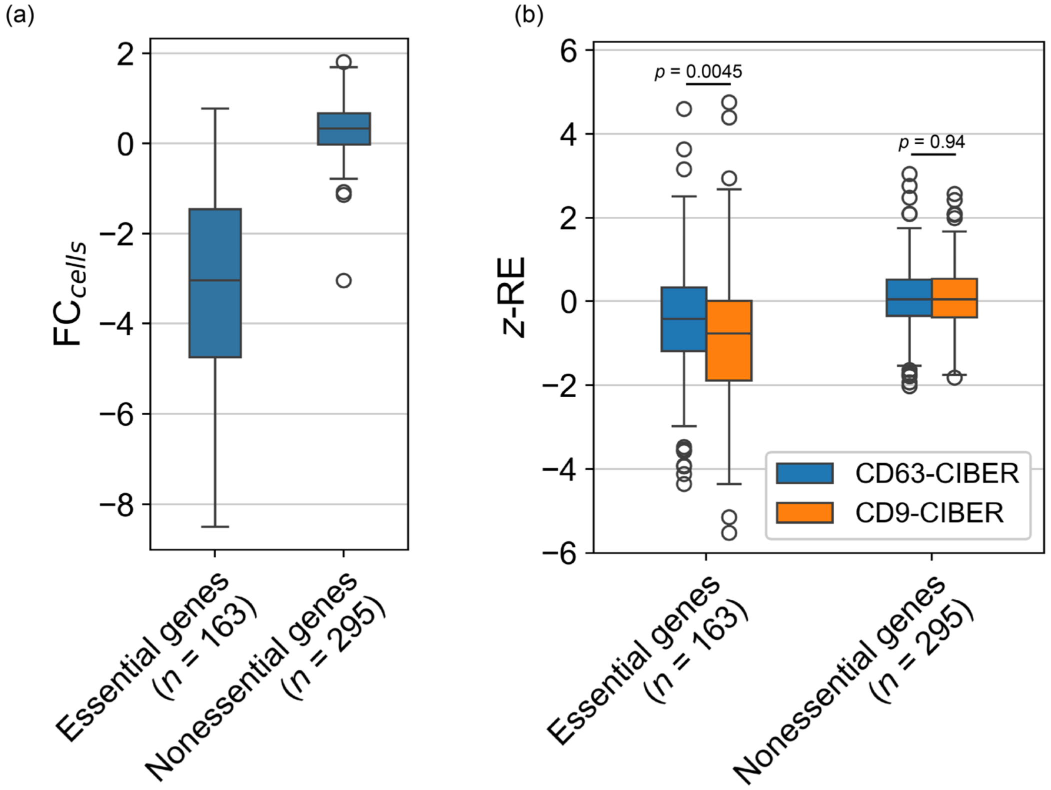

Supplementary Figure 30 | *z*-RE scores of genes essential for cell survival (Essential genes) and genes not essential for cell survival (Nonessential genes) (see Source Data for the list of genes in each group) in CD63-CIBER and CD9-CIBER in HEK293T cells. Box plots show the distribution of FC_{cells} in CD63-CIBER (a) and *z*-REs across CD63-CIBER and CD9-CIBER (b). The central line within each box represents the median, the edges of the box indicate the interquartile range (IQR, 25th to 75th percentile), and the whiskers extend to the smallest and largest values within 1.5 times the IQR from the quartiles. Outliers are shown as individual open circle beyond the whiskers. The result shows that KO of essential genes dominantly suppresses the release of $CD9^+$ sEVs, which might indicate that cell division itself (rather than key regulator genes) influences the release of $CD9^+$ sEVs. *p*: two-tailed Welch's *t*-test.

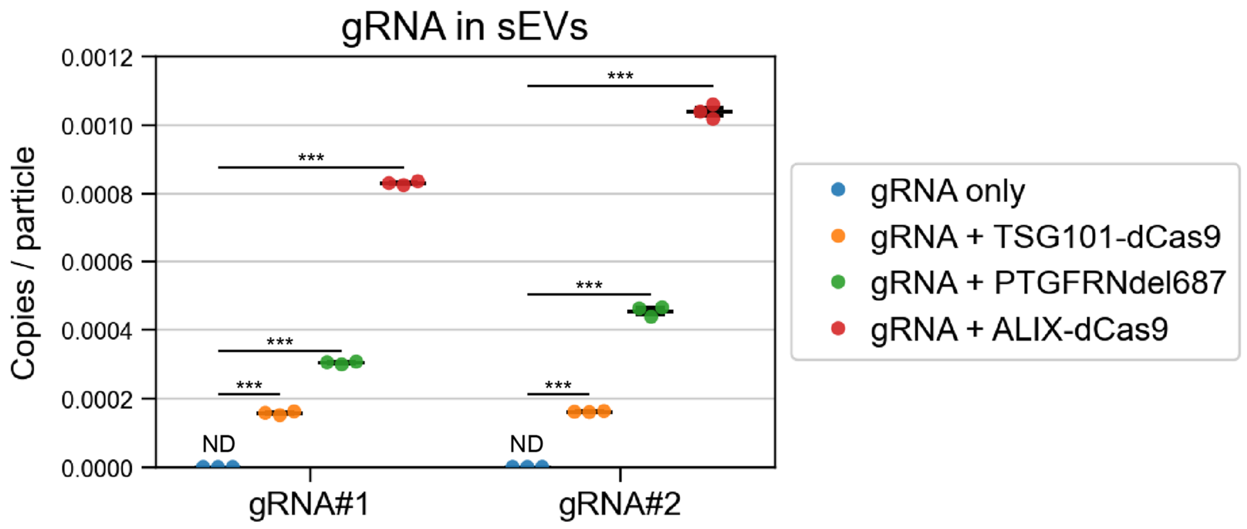

Supplementary Figure 31 | gRNA abundance in sEVs isolated from culture media of HEK293T or HEK293T cells expressing dCas9-fused EV marker (TSG101, PTGFRNΔ687[12] and ALIX) stably transduced with gRNA using lentivirus. The copy numbers of gRNAs were determined by qPCR (using gRNA-encoding plasmid as a standard) and divided by the particle number measured by Nanosight to calculate copies/particle. The Ct values of qPCR for gRNA#1 and #2 under gRNA only conditions were not significantly different from the no-gRNA control, so the copy numbers are shown as ND (not determined). p: one sample *t*-test under the null hypothesis where copies/particle equals 0 with Holm correction. Error bars represent ± SEM of biological replicates (n = 3).

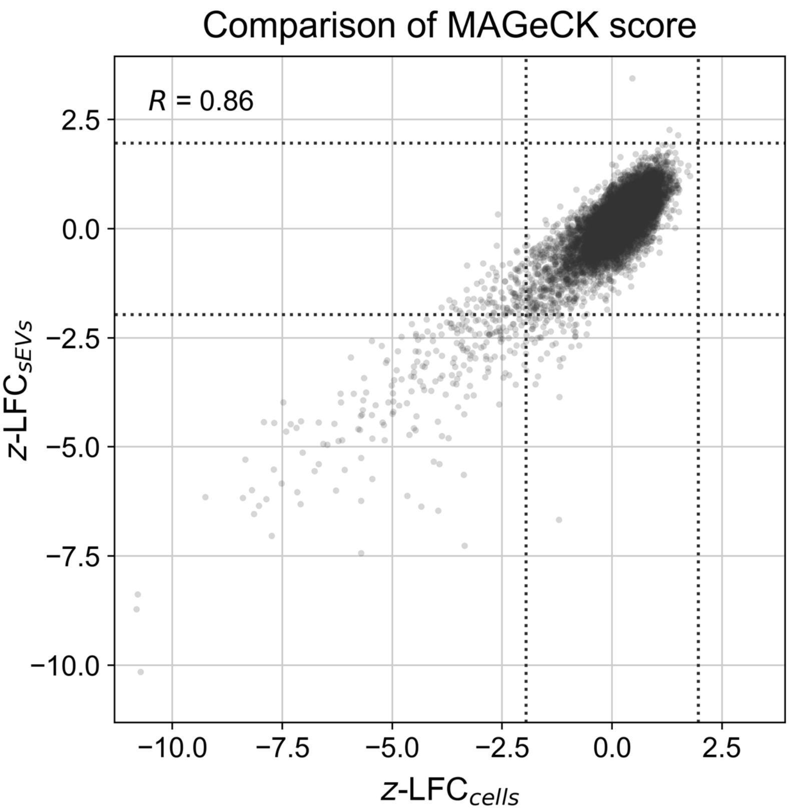

Supplementary Figure 32 | Data showing the applicability of the sEV-barcoding system for cell-free CRISPR screening. The pair-wise plot of *z*-normalized $\log_2$ fold-change of the MAGeCK scores[22] of 9,936 genes (z-LFC, Cas9+ vs Cas9-) are shown. The scores are calculated from gRNA abundance in sEVs and cells with the data of CD63-CIBER screening (excluding the hit genes of the CD63-CIBER screening; i.e. CIBER screening was used as the counter assay). The numbers in each region divided by dashed lines show the number of genes in it. z-LFC_{cells} is almost equivalent to the output of normal CRISPR screening, reflecting the changes of the cell population resulting from gene KO. Three hundred and sixteen genes were detected as lower hits under a threshold of +/- 1.96, an empirical threshold for selecting the top/bottom 5% of the population. z-LFC_{cells} and z-LFC_{sEVs} were highly correlated (R = 0.87) and the cell-free output obtained by sequencing barcodes in sEVs (z-LFC_{sEVs}) successfully detected 231 genes out of 316 hit genes calculated from the cellular barcodes. Dashed lines show +/-1.96.

Supplementary references

1. Kowarz, E., Löscher, D. & Marschalek, R. Optimized Sleeping Beauty transposons rapidly generate stable transgenic cell lines. *Biotechnol. J.* **10**, 647–653 (2015).
2. Horlbeck, M. A. *et al.* Compact and highly active next-generation libraries for CRISPR-mediated gene repression and activation. *Elife* **5**, e19760 (2016).
3. Wroblewska, L. *et al.* Mammalian synthetic circuits with RNA binding proteins for RNA-only delivery. *Nat. Biotechnol.* **33**, 839–841 (2015).
4. Konermann, S. *et al.* Genome-scale transcriptional activation by an engineered CRISPR-Cas9 complex. *Nature* **517**, 583–588 (2015).
5. Kojima, R. *et al.* Designer exosomes produced by implanted cells intracerebrally deliver therapeutic cargo for Parkinson's disease treatment. *Nat. Commun.* **9**, 1305 (2018).
6. Ho, S.-M. *et al.* Evaluating Synthetic Activation and Repression of Neuropsychiatric-Related Genes in hiPSC-Derived NPCs, Neurons, and Astrocytes. *Stem Cell Reports* **9**, 615–628 (2017).
7. Sanjana, N. E., Shalem, O. & Zhang, F. Improved vectors and genome-wide libraries for CRISPR screening. *Nat. Methods* **11**, 783–784 (2014).
8. Morgens, D. W. *et al.* Genome-scale measurement of off-target activity using Cas9 toxicity in high-throughput screens. *Nat. Commun.* **8**, 15178 (2017).
9. Montague, T. G., Cruz, J. M., Gagnon, J. A., Church, G. M. & Valen, E. CHOPCHOP: a CRISPR/Cas9 and TALEN web tool for genome editing. *Nucleic Acids Res.* **42**, W401-7 (2014).
10. Tanenbaum, M. E., Gilbert, L. A., Qi, L. S., Weissman, J. S. & Vale, R. D. A protein-tagging system for signal amplification in gene expression and fluorescence imaging. *Cell* **159**, 635–646 (2014).
11. Bindels, D. S. *et al.* mScarlet: a bright monomeric red fluorescent protein for cellular imaging. *Nat. Methods* **14**, 53–56 (2017).
12. Dooley, K. *et al.* A versatile platform for generating engineered extracellular vesicles with defined therapeutic properties. *Mol. Ther.* **29**, 1729–1743 (2021).
13. Livak, K. J. & Schmittgen, T. D. Analysis of relative gene expression data using real-time quantitative PCR and the 2(-Delta Delta C(T)) Method. *Methods* **25**, 402–408 (2001).
14. Lu, A. *et al.* Genome-wide interrogation of extracellular vesicle biology using barcoded miRNAs. *Elife* **7**, e41460 (2018).
15. Gross, J. C., Chaudhary, V., Bartscherer, K. & Boutros, M. Active Wnt proteins are secreted on exosomes. *Nat. Cell Biol.* **14**, 1036–1045 (2012).
16. Colombo, M., Raposo, G. & Théry, C. Biogenesis, secretion, and intercellular interactions of exosomes and other extracellular vesicles. *Annu. Rev. Cell Dev. Biol.* **30**, 255–289 (2014).
17. Pathan, M. *et al.* Vesiclepedia 2019: a compendium of RNA, proteins, lipids and metabolites in extracellular vesicles. *Nucleic Acids Res.* **47**, D516–D519 (2019).
18. Kalra, H. *et al.* Vesiclepedia: a compendium for extracellular vesicles with continuous community annotation. *PLoS Biol.* **10**, e1001450 (2012).

19. Armstrong, J. S., Hornung, B., Lecane, P., Jones, D. P. & Knox, S. J. Rotenone-induced G2/M cell cycle arrest and apoptosis in a human B lymphoma cell line PW. *Biochem. Biophys. Res. Commun.* **289**, 973–978 (2001).
20. Schwanhäusser, B. *et al.* Global quantification of mammalian gene expression control. *Nature* **473**, 337–342 (2011).
21. Joung, J. *et al.* Genome-scale CRISPR-Cas9 knockout and transcriptional activation screening. *Nat. Protoc.* **12**, 828–863 (2017).
22. Li, W. *et al.* MAGeCK enables robust identification of essential genes from genome-scale CRISPR/Cas9 knockout screens. *Genome Biol.* **15**, 554 (2014).

Uncropped Scans

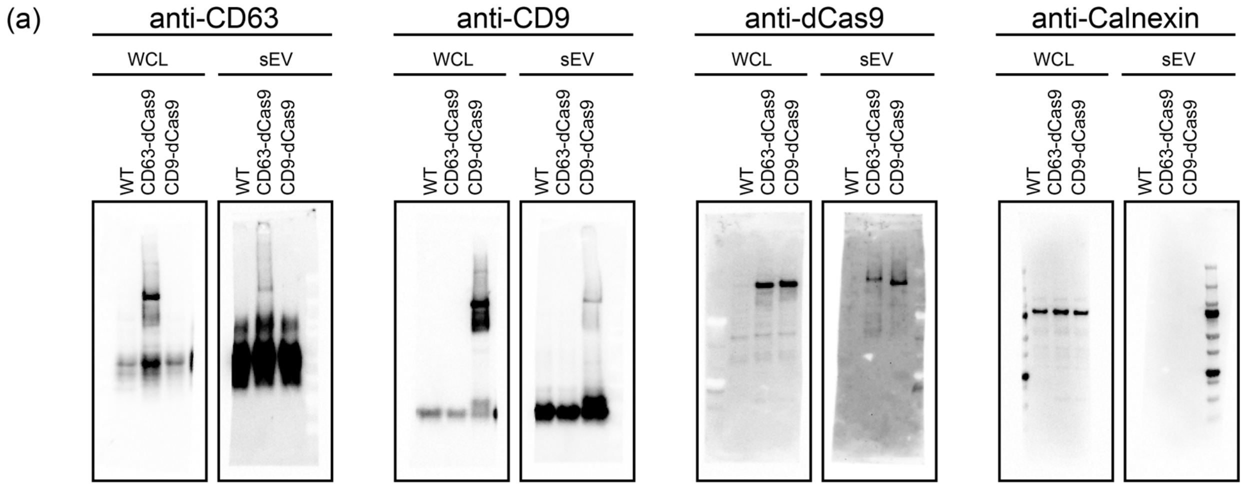

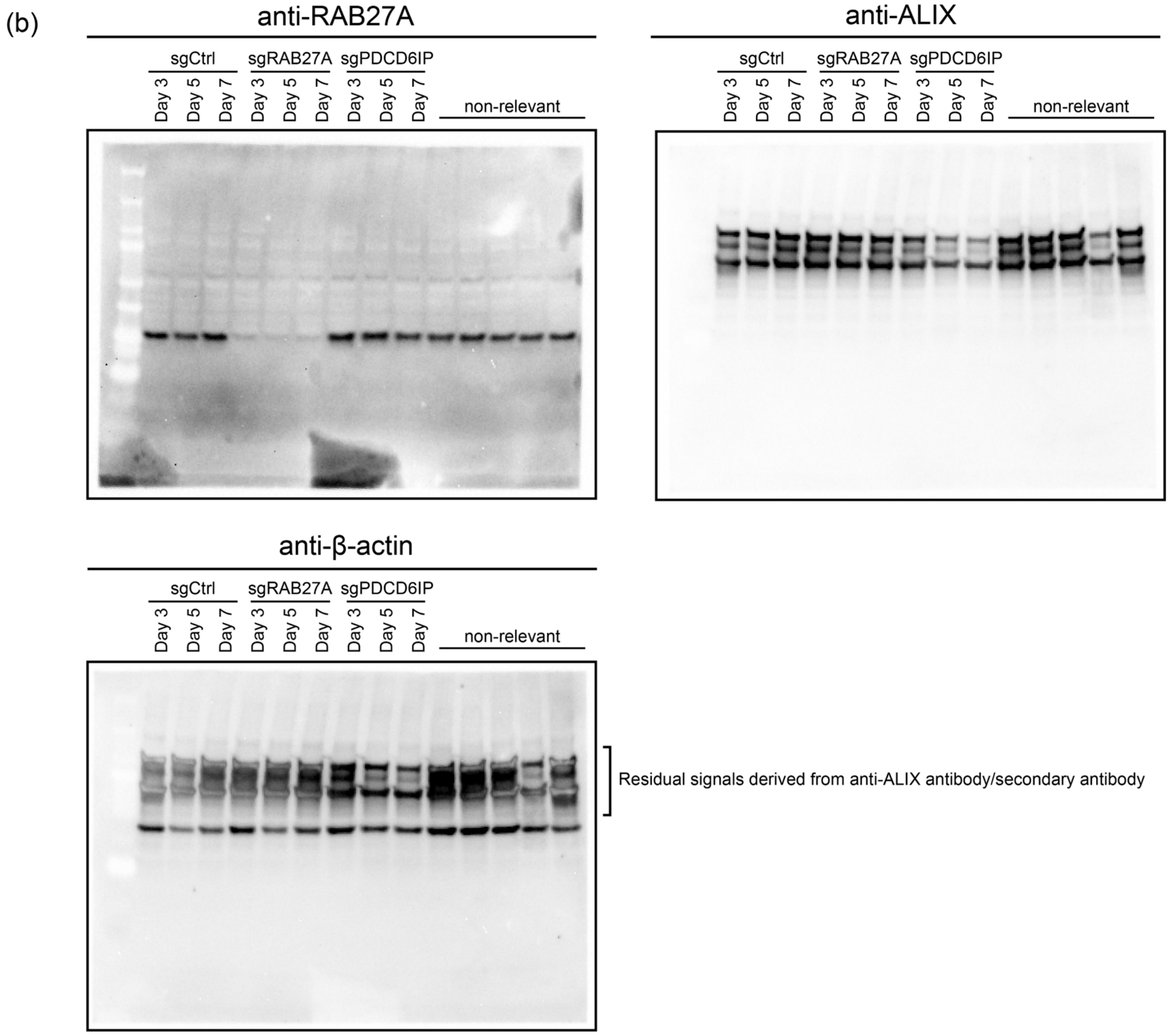

Uncropped scans for Supplementary Fig. 2a (a) and 28c (b). Membranes are prepared for each antibody for (a). A single membrane was used for 3 rounds of probing for (b).
