# Supplementary material for: Barcoding of small extracellular vesicles with CRISPR-gRNA enables comprehensive, subpopulation-specific analysis of their biogenesis and release regulators
Source: Nat Commun. 2024 Nov 19;15:9777. doi: 10.1038/s41467-024-53736-x (PMC11577021; doi:10.1038/s41467-024-53736-x)
Supplement: Supplementary file 2 — Description of Additional Supplementary Information [file 41467_2024_53736_MOESM2_ESM.docx]

**Description of Additional Supplementary Files**

File Name: Supplementary Data 1

Description: Sequence metrics of the NGS data of CD63 and CD9 CIBER screening

File Name: Supplementary Data 2

Description: Read Count of each gRNA in CIBER screening

File Name: Supplementary Data 3

Description: FCcells and FCsEVs of gRNAs in CIBER screening

File Name: Supplementary Data 4

Description: zRE of genes in CIBER screening

File Name: Supplementary Data 5

Description: Results of GO analysis of CD63-CIBER and CD9-CIBER in HEK293T cells

File Name: Supplementary Data 6

Description: Results of GSEAPreranked of CD63-CIBER and CD9-CIBER in HEK293T cells

File Name: Supplementary Data 7

Description: Spacer sequences of the gRNA used

File Name: Supplementary Data 8

Description: Results of TF-IDF analysis of CD63-CIBER and CD9-CIBER

File Name: Supplementary Data 9

Description: Read Count and TPM of RNA-seq regarding Suppl Fig 28
