# Supplementary material for: Barcoding of small extracellular vesicles with CRISPR-gRNA enables comprehensive, subpopulation-specific analysis of their biogenesis and release regulators
Source: Nat Commun. 2024 Nov 19;15:9777. doi: 10.1038/s41467-024-53736-x (PMC11577021; doi:10.1038/s41467-024-53736-x)
Supplement: Supplementary file 12 — Reporting Summary [file 41467_2024_53736_MOESM12_ESM.pdf]

nature portfolio

Corresponding author(s): Ryosuke Kojima

Last updated by author(s): Oct 17, 2024

Reporting Summary

Nature Portfolio wishes to improve the reproducibility of the work that we publish. This form provides structure for consistency and transparency in reporting. For further information on Nature Portfolio policies, see our Editorial Policies and the Editorial Policy Checklist.

Statistics

For all statistical analyses, confirm that the following items are present in the figure legend, table legend, main text, or Methods section.

n/a	Confirmed	
☐	☒	The exact sample size (*n*) for each experimental group/condition, given as a discrete number and unit of measurement
☐	☒	A statement on whether measurements were taken from distinct samples or whether the same sample was measured repeatedly
☐	☒	The statistical test(s) used AND whether they are one- or two-sided<br>*Only common tests should be described solely by name; describe more complex techniques in the Methods section.*
☒	☐	A description of all covariates tested
☐	☒	A description of any assumptions or corrections, such as tests of normality and adjustment for multiple comparisons
☐	☒	A full description of the statistical parameters including central tendency (e.g. means) or other basic estimates (e.g. regression coefficient) AND variation (e.g. standard deviation) or associated estimates of uncertainty (e.g. confidence intervals)
☐	☒	For null hypothesis testing, the test statistic (e.g. *F*, *t*, *r*) with confidence intervals, effect sizes, degrees of freedom and *P* value noted<br>*Give P values as exact values whenever suitable.*
☒	☐	For Bayesian analysis, information on the choice of priors and Markov chain Monte Carlo settings
☐	☒	For hierarchical and complex designs, identification of the appropriate level for tests and full reporting of outcomes
☐	☒	Estimates of effect sizes (e.g. Cohen's *d*, Pearson's *r*), indicating how they were calculated

Our web collection on statistics for biologists contains articles on many of the points above.

Software and code

Policy information about availability of computer code

Data collection: N/A

Data analysis: We conducted data analysis as follows as mentioned in the manuscript.

Unless otherwise stated, all computational and statistical analyses in this study were performed using Excel, Python or R.
Protein-protein interaction was done by STRING database via the StringApp plugin (https://apps.cytoscape.org/apps/stringapp) on Cytoscape49 (ver. 3.9.1, https://cytoscape.org/).
Enrichment analysis against the GO Biological Process data set was performed using the ClueGO plugin (ver. 2.5.9, https://apps.cytoscape.org/apps/cluego) on Cytoscape (ver. 3.9.1).
GSEA was performed with GSEA software (ver. 4.2.3, https://www.gsea-msigdb.org/gsea/index.jsp)
We share python scripts used in this study via GitHub: https://github.com/Ryosuke-Kojima/CIBER-screening-paper

For manuscripts utilizing custom algorithms or software that are central to the research but not yet described in published literature, software must be made available to editors and reviewers. We strongly encourage code deposition in a community repository (e.g. GitHub). See the Nature Portfolio guidelines for submitting code & software for further information.

Data

Policy information about availability of data

All manuscripts must include a data availability statement. This statement should provide the following information, where applicable:

- Accession codes, unique identifiers, or web links for publicly available datasets
- A description of any restrictions on data availability
- For clinical datasets or third party data, please ensure that the statement adheres to our policy

All data generated in this study is available in the Source Data, Supplementary Data, and public repositories (DDBJ database (under accession code, PRJDB17057, https://ddbj.nig.ac.jp/search/entry/bioproject/PRJDB17057). Sequence data of relevant plasmids have been deposited in GenBank: pKK60 (PQ146490), pRK300 (PQ146491), pKK106 (PQ146492), pKK108 (PQ146493), pKK147 (PQ146494), pKK272 (PQ146495), pKK273 (PQ146496), pKK274 (PQ146497).

Research involving human participants, their data, or biological material

Policy information about studies with human participants or human data. See also policy information about sex, gender (identity/presentation), and sexual orientation and race, ethnicity and racism.

Reporting on sex and gender	N/A
Reporting on race, ethnicity, or other socially relevant groupings	N/A
Population characteristics	N/A
Recruitment	N/A
Ethics oversight	N/A

Note that full information on the approval of the study protocol must also be provided in the manuscript.

Field-specific reporting

Please select the one below that is the best fit for your research. If you are not sure, read the appropriate sections before making your selection.

☒ Life sciences ☐ Behavioural & social sciences ☐ Ecological, evolutionary & environmental sciences

For a reference copy of the document with all sections, see nature.com/documents/nr-reporting-summary-flat.pdf

Life sciences study design

All studies must disclose on these points even when the disclosure is negative.

Sample size	All the sample size was indicated in the figure legends. Sample size was generally chosen on our experience and the standard practices in the field (typically 3 or more biological replicates for biochemical assays). No statistical methods were used to determine the sample size.
Data exclusions	In general, all the data were included. For the NGS-based barcode counting, barcodes with normalized read count lower than 0.05 in at least one sample were excluded from downstream calculation due to the unreliability of the data. And after this exclusion, any genes with less than 3 barcodes were also excluded for the robust analysis. This criteria was set before the analysis. These data processing procedures are described in the Methods section.
Replication	All the experimental findings were reliably reproduced.
Randomization	Cell lines were used throughout this study, so bias of the results due to the heterogeneity of the analytes should be minimum. So we did not conduct randomization.
Blinding	It was practically impossible to do blinding in each experiment due to the high labor costs. No aminal or human research participants were used and the results should be rather stable, so we believe there was no big issue about blinding for group allocation in this study.

Reporting for specific materials, systems and methods

We require information from authors about some types of materials, experimental systems and methods used in many studies. Here, indicate whether each material, system or method listed is relevant to your study. If you are not sure if a list item applies to your research, read the appropriate section before selecting a response.

Materials & experimental systems

n/a	Involved in the study
☐	☒ Antibodies
☐	☒ Eukaryotic cell lines
☒	☐ Palaeontology and archaeology
☒	☐ Animals and other organisms
☒	☐ Clinical data
☒	☐ Dual use research of concern
☒	☐ Plants

Methods

n/a	Involved in the study
☒	☐ ChIP-seq
☐	☒ Flow cytometry
☒	☐ MRI-based neuroimaging

Antibodies

Antibodies used

(ELISA) Anti-CD63 antibody was supplied with PS Capture Exosome ELISA Kit (cat. #298-80601, Fujifilm) and CD63-Capture Human Exosome ELISA Kit (cat. #290-83601, Fujifilm). Anti-CD9 antibody was supplied with CD9-Capture Human Exosome ELISA Kit (cat. #296-83701, Fujifilm). Antibodies were used according to the manufacturer's protocol. For the detection of CD9 on sEV surface by PS Capture Exosome ELISA Kit, Anti-CD9 antibody was purchased from Fujifilm (cat. #013-27951) and used at 240 ng/mL diluted with Reaction Buffer included in the kit.

(Western blotting) Anti-CD63 antibody (1:100, cat. #SHI-EXO-M02, Lot 23H23CB, CosmoBio), anti-CD9 antibody (1:100, cat. #SHI-EXO-M01, lot 28G23CB, CosmoBio), anti-dCas9 antibody (1:100, cat. #A-9000-010, lot 2203061, Epigen Tek), anti-calnexin antibody (1:1000, cat. #EPR3633(2), lot GR3416744-20, abcam), anti-RAB27A antibody (1:500, cat. #95394, lot 1, Cell Signaling Technology), anti-ALIX antibody (1:1000, cat. #12422-1-AP, lot 00115246, Proteintech), anti-β-actin antibody (1:1000, cat. #4970, lot 19, Cell Signaling Technology), HRP-conjugated secondary antibody (1:1000, cat. #7074, lot 33 or #7076, lot 38, Cell Signaling Technology)

(Immunofluorescence) Anti-CD63 antibody (1:100, cat. #SHI-EXO-M02, Lot 23H23CB, CosmoBio), anti-CD9 antibody (1:100, cat. #SHI-EXO-M01, lot 28G23CB, CosmoBio), anti-dCas9 antibody (1:100, cat. #A-9000-010, lot 2203061, Epigen Tek), anti-GFP antibody (1:500, cat. #598, MBL), secondary antibody (1:400, cat. #A32740, lot YI378038 or #A32723, lot YJ383140 Invitrogen)

Validation

(ELISA) Antibodies were validated by manufacturer. Information of the ELISA kit can be found here: https://labchem-wako.fujifilm.com/us/product/detail/W01W0129-8060.html, https://labchem-wako.fujifilm.com/jp/product/detail/W01W0129-8360.html, https://labchem-wako.fujifilm.com/jp/product/detail/W01W0129-8370.html. Information of the anti-CD9 antibody is available here: https://labchem-wako.fujifilm.com/jp/product/detail/W01W0101-2795.html.

(Western blotting) Antibodies were validated by confirming that the antibodies recognizes the correct-sized band. Additionally, anti-CD63, CD9 and dCas9 antibodies were validated by the stronger signal from positive control samples over-expressing the target protein. Anti calnexin antibody was validated by confirming the absence of calnexin in negative control sample (sEV). Anti-RAB27A and ALIX antibody were validated to ensure that the amount of target protein is reduced in knock out cell lines.

(Immunofluorescence) Anti-CD63, CD9 and dCas9 antibodies were validated in western blotting experiments and also by confirming that the antibodies stained the expected cellular localization of the target protein in wild type cells. Anti-GFP antibody was validated by the stronger signal from positive control cell lines over-expressing GFP.

All antibodies were used in applications for which the manufacturers indicated they were suitable.

Eukaryotic cell lines

Policy information about cell lines and Sex and Gender in Research

Cell line source(s)	HEK293T cells were distributed by RIKEN BRC CELL BANK (RCB2202) (Tsukuba, Japan). SH-SY5Y cells were kind gift from Dr. Yukiko Hori (originally from ATCC (VA, USA, CRL-2266)), HT29 cells were purchased from ATCC (VA, USA, HTB-38).
Authentication	The provider of the cells conduct the quality control of the cells by methods described in their website. The cells utilized in this study have been used in many publications.
Mycoplasma contamination	The provider of the cells confirm that the cells are Mycoplasma free. Also, we routinely check Mycoplasma contamination by MycoAlert (Lonza) or Takara PCR Mycoplasma Detection Set (Takara)
Commonly misidentified lines (See ICLAC register)	N/A

Plants

Seed stocks

N/A

Novel plant genotypes

Describe the methods by which all novel plant genotypes were produced. This includes those generated by transgenic approaches, gene editing, chemical/radiation-based mutagenesis and hybridization. For transgenic lines, describe the transformation method, the number of independent lines analyzed and the generation upon which experiments were performed. For gene-edited lines, describe the editor used, the endogenous sequence targeted for editing, the targeting guide RNA sequence (if applicable) and how the editor was applied.

Authentication

Describe any authentication procedures for each seed stock used or novel genotype generated. Describe any experiments used to assess the effect of a mutation and, where applicable, how potential secondary effects (e.g. second site T-DNA insertions, mosiacism, off-target gene editing) were examined.

Flow Cytometry

Plots

Confirm that:

- [x] The axis labels state the marker and fluorochrome used (e.g. CD4-FITC).
- [x] The axis scales are clearly visible. Include numbers along axes only for bottom left plot of group (a 'group' is an analysis of identical markers).
- [x] All plots are contour plots with outliers or pseudocolor plots.
- [x] A numerical value for number of cells or percentage (with statistics) is provided.

Methodology

Sample preparation

The method is described in the Methods section, Double Thymidine block (in main text) and Flow cytometry analysis of knockout efficiency (in Supplementaty information)

Instrument

BD LSR II (Becton, Dickinson and Company)

Software

FCM data was processed on FACS Diva software (ver. 4.1) and further analyzed by using a Python package FlowCal51 (https://taborlab.github.io/FlowCal/)

Cell population abundance

Cell sorting was not conducted.

Gating strategy

Cells were separated from debris and dead cells by FSC-A/SSC-A gate generated around the region with the highest density to retain approximately 90% of the primary population. After that, doublets were removed by FFC-W/FFC-H gate where plots with exceptionally higher width/hight ratio were considered to be doublets.

(Flow cytometry analysis of knockout efficiency) The 99th percentile of BFP-negative cells were 1,500 so 1,500 were defined as boundary between BFP-positive and BFP-negative cells.

(Double Thymidine block) The G1 peak appeared around 45,000 < DNA dye fluorescence < 50,000 and the G2/M peak appeared around 85,000 < DNA dye fluorescence < 90,000, so we assigned cells with DNA dye fluorescence larger than 80,000 to M phase.

- [x] Tick this box to confirm that a figure exemplifying the gating strategy is provided in the Supplementary Information.
